# Mechanical Characterization of the Vessel Wall by Data Assimilation of Intravascular Ultrasound Studies

**DOI:** 10.3389/fphys.2018.00292

**Published:** 2018-03-28

**Authors:** Gonzalo D. Maso Talou, Pablo J. Blanco, Gonzalo D. Ares, Cristiano Guedes Bezerra, Pedro A. Lemos, Raúl A. Feijóo

**Affiliations:** ^1^National Laboratory for Scientific Computing, Department of Mathematical and Computational Methods, Petrópolis, Brazil; ^2^National Institute of Science and Technology in Medicine Assisted by Scientific Computing, São Paulo, Brazil; ^3^National Scientific and Technical Research Council, Buenos Aires, Argentina; ^4^CAE Group, National University of Mar del Plata, Mar del Plata, Argentina; ^5^Department of Interventional Cardiology, Heart Institute (Incor), São Paulo, Brazil

**Keywords:** parameter identification, reduced order unscented Kalman filter, IVUS, coronary arteries, arterial wall model, computational models, high performance computing

## Abstract

Atherosclerotic plaque rupture and erosion are the most important mechanisms underlying the sudden plaque growth, responsible for acute coronary syndromes and even fatal cardiac events. Advances in the understanding of the culprit plaque structure and composition are already reported in the literature, however, there is still much work to be done toward *in-vivo* plaque visualization and mechanical characterization to assess plaque stability, patient risk, diagnosis and treatment prognosis. In this work, a methodology for the mechanical characterization of the vessel wall plaque and tissues is proposed based on the combination of intravascular ultrasound (IVUS) imaging processing, data assimilation and continuum mechanics models within a high performance computing (HPC) environment. Initially, the IVUS study is gated to obtain volumes of image sequences corresponding to the vessel of interest at different cardiac phases. These sequences are registered against the sequence of the end-diastolic phase to remove transversal and longitudinal rigid motions prescribed by the moving environment due to the heartbeat. Then, optical flow between the image sequences is computed to obtain the displacement fields of the vessel (each associated to a certain pressure level). The obtained displacement fields are regarded as observations within a data assimilation paradigm, which aims to estimate the material parameters of the tissues within the vessel wall. Specifically, a reduced order unscented Kalman filter is employed, endowed with a forward operator which amounts to address the solution of a hyperelastic solid mechanics model in the finite strain regime taking into account the axially stretched state of the vessel, as well as the effect of internal and external forces acting on the arterial wall. Due to the computational burden, a HPC approach is mandatory. Hence, the data assimilation and computational solid mechanics computations are parallelized at three levels: (i) a Kalman filter level; (ii) a cardiac phase level; and (iii) a mesh partitioning level. To illustrate the capabilities of this novel methodology toward the *in-vivo* analysis of patient-specific vessel constituents, mechanical material parameters are estimated using *in-silico* and *in-vivo* data retrieved from IVUS studies. Limitations and potentials of this approach are exposed and discussed.

## 1. Introduction

Cardiovascular diseases are the principal cause of death and morbidity worldwide (Mathers et al., 2016). The two principal causes of death, cardiac ischemia and stroke, are intrinsically related with the onset and progress and destabilization processes of atherosclerotic plaque, which are still largely unknown (Crea and Liuzzo, 2013; Bentzon et al., 2014). At the final stage of the destabilization process, the plaque ruptures releasing thrombotic components into the blood stream which in turn generate thrombi that block the vessel lumen causing ischemia. Thus, the prediction of rupture events and the identification of the so-called culprit plaques is of the utmost importance for diagnostics and therapeutics. Through computational simulations is it possible to study the arterial wall stress state, which may compromise plaque integrity and induce rupture. Moreover, computational models also allow to recreate different physiological and pathophysiological conditions (hypertension, hyperemia, exercise, stenosis) (Taylor et al., 1999, 2013; Torii et al., 2007; Blanco et al., 2015), as well as interventions (angioplasty balloon inflation, stent deployment, stent-plaque interaction, among others) (Conway et al., 2012, 2014) that are valuable resources for diagnosis, treatment and surgical risk assessment.

In order to accurately simulate patient specific conditions, three kinds of input data are required: (i) patient-specific anatomical models of the vasculature, (ii) the loads to which the anatomical structures are subjected to, and (iii) the patient-specific distribution of the arterial-wall constituents and their corresponding material parameters. As far as anatomical data of the arteries is concerned, it can be straighforwardly extracted from different medical imaging modalities (Wahle et al., 1995; Milner et al., 1998; Bulant et al., 2017). Regarding the force exerted by the blood pressure, it can be accurately estimated from cuff-pressure measurements (O'brien et al., 2001; Miyashita, 2012). Thus, we are left to the problem of setting patient-specific material parameters for the models of the arterial wall. This has long been the Achilles tendon in numerical simulations, most of them relying in material parameters acquired from *ex-vivo* material experimentation in cadaveric specimens (Walsh et al., 2014; Karimi et al., 2015). In this sense, the *in-vivo* identification of material parameters for the arterial-wall is still an open research topic.

Toward covering the aforementioned gap, specifically in the coronary artery disease domain, intravascular ultrasound (IVUS) emerges as an suitable imaging modality to make the attempt to retrieve the material parameters and distribution of the vessel materials under *in-vivo* conditions due to its high temporal and spatial resolution. The acquired images, when coherently ordered, are capable of delivering the motion of the vascular structures. Some works (Kawasaki et al., 2002; Nair et al., 2002; Sathyanarayana et al., 2009) have successfully classified the materials in few discrete categories (e.g., necrotic core, fibrotic, fibro-fatty or lipid-pool, calcified) based on the acoustic impedance response of the tissues in a determined frame of the IVUS study. It has then been demonstrated that there is a notorious variability of the stress-strain response of tissues within the same category (Loree et al., 1994; Holzapfel et al., 2005; Walsh et al., 2014) of such classification. Therefore, this information is not specific enough for simulation purposes. As anticipated above, the temporal resolution of the IVUS study can be exploited to retrieve the motion (displacement field) of the vessel wall along the cardiac cycle (for example by using optical flow techniques or large deformation diffeomorphic metric mapping). Using the displacement field as input, data assimilation techniques can be supplied to estimate the material parameters.

Data assimilation techniques make use of measurable quantities to adjust a physical model whose goal is to represent the reality posed by the *in-vivo* scenario. In that manner, these techniques permit not only to estimate specific quantities of interest, but also to explore the underlying physical phenomena. Also, measurement errors can be filtered by the physical model being a *quid pro quo* benefit: the measurements instantiate the model and the model filters the measurements. Such techniques can be classified in two categories: (i) variational approaches and (ii) sequential filtering approaches.

In the variational approach, a cost functional that measures the difference between the observed measures and the model prediction is constructed. The cost functional depends on the parameters of interest (among other parameters required by the model) to render a model prediction of the measured variable. Then, the estimated parameters are those such that minimize the cost functional. The more popular approach is to solve the Karush-Kuhn-Tucker (KKT) necessary conditions which is employed in several works for mechanical parameter estimation (Lagrée, 2000; Martin et al., 2005; Sermesant et al., 2006; Perego et al., 2011; D'Elia et al., 2012; Bertagna and Veneziani, 2014; Ares, 2016). In Lagrée (2000), the viscoelastic parameters of large arteries were estimated using displacement fields of the vessel wall generated by computational models. Similarly, Martin et al. (2005) explored the estimation of the vessel compliance in a 1D model using a 3D fluid-structure interaction (FSI) model to generate the measured displacement of the vessel wall. Using medical data of blood pressure and inner radius of the arteries, Stålhand (2009) also used 1D models to estimate the material parameters according to the model proposed in Holzapfel et al. (2000). The works of Perego et al. (2011) and D'Elia et al. (2012) formulate the inverse problem from 3D FSI models and analyze the sensitivity in the identification of Young modulus to noise in the measurements of arterial wall displacements. In the latter, data assimilation is performed from flow velocity data as well. The main drawback of these variational approaches is the large number of evaluations of the cost functional (or its derivative) which are required in the minimization problem (Lassila et al., 2013). Furthermore, the use of more realistic models such as 3D FSI models or complex heterogeneous anisotropic solid models are many times mandatory to render accurate results, increasing the computational effort. In some cases, reduced order strategies combined with statistical approaches can be applied to reduce the burden behind cost functional evaluations, as shown in Lassila et al. (2013). Other approach is proposed in Bertagna and Veneziani (2014), based on the application of model reduction techniques coupled with a proper orthogonal decomposition to accomplish the solution of 3D FSI in a computationally efficient way. Efficient implementations for solid mechanics problems have also been proposed in Avril et al. (2010) and Pérez Zerpa and Canelas (2016) using a virtual fields method and a constitutive equation gap functional, respectively.

In turn, and for problems involving a small-to-moderate number of unknown parameters, the sequential filtering approach (also known as filtering methods) is less computationally demanding and, at the same time, embarrassingly parallel. These features make the filtering approach an appealing strategy for the kind of problems addressed in the present work. Conceptually, given a set of observations, the method realizes a prediction for each observation and, then, introduces corrections in the model parameters based on the discrepancies between the model estimation and the observed data. For each prediction-correction step, several variations of the parameters are tested in the model and, through statistical analysis of the model predictions, a suitable correction is performed over the parameters. Several methods based on the Kalman filter have been developed to deal with linear and non-linear dynamic problems. As examples, a non-linear extended Kalman filter (EKF) with collocation feedback is applied to identify the Young modulus of different regions of a heart model in Moireau et al. (2008), Moireau et al. (2009), and Chapelle et al. (2009). The observations used varied between the myocardium velocity (Moireau et al., 2008), displacement (Moireau et al., 2009) and velocity of the heart boundaries (Chapelle et al., 2009). The stability of such methods was studied (Moireau et al., 2008) and in terms of accuracy it is reported that Kalman filtering is optimal for linear systems only, while extended algorithms based on linearized operators may lead to efficient, albeit non-optimal, filtering procedures. In Lipponen et al. (2010), the EKF is also applied to estimate parameters of a reduced order Navier-Stokes model (through an orthogonal decomposition of the velocity field) through observations acquired from electrical impedance tomography. In more recent works, Moireau and Chapelle (2011) presented a reduced order Kalman filter based on the unscented transform (abbreviated as ROUKF) that offers an interesting alternative to the EKF method. Such an approach does not require neither linearization nor calculation of the tangent operator of the non-linear model, which substantially eases its implementation. Noteworthy, the ROUKF features a higher order approximation of the system states statistics, delivering more accurate outcomes than EKF. In Bertoglio et al. (2012) and Bertoglio et al. (2014), ROUKF was successfully applied for estimation of Young modulus in arteries with tests *in-vivo* and *in-vitro*, showing a simpler and more efficient implementation than EKF. Recently in Caiazzo et al. (2017), terminal resistances and vessel wall properties of a 1D vascular network were estimated via ROUKF using blood flow and/or pressure measurements as observations.

In this work, we present a novel approach to construct patient-specific mechanical models of the arterial wall using *in-vivo* data from IVUS studies. In a nutshell, this approach integrates the realms of image processing, optical flow, continuum mechanics, and filtering data assimilation to effectively merge patient-specific data with mechanical models, toward the *in-vivo* estimation of material properties. From the IVUS study, a frame of interest is selected and the corresponding arterial wall is demarcated. For the mechanical model a finite strain framework is considered, and the constituent tissues are assumed to behave as isotropic Neo-Hookean materials. Importantly, it is considered that the arterial vessel corresponding to the diastolic phase is at equilibrium with a certain diastolic pressure level, and it is further subjected to a given axial stretch at that phase. By using gating, registration and optical flow methods developed in previous works (Maso Talou et al., 2015, 2017; Maso Talou, 2017), the displacement field of the vessel wall is estimated along the cardiac cycle. Then, the ROUKF is exploited as a data assimilation procedure in which the previously obtained displacement field is supplied as observational data, while the material parameter of the Neo-Hookean models are the target parameters to be estimated.

The manuscript is structured as follows. In section 2, the proposed methodology is detailed, presenting image processing techniques (section 2.1), the mechanical model for the arterial wall (section 2.2), and, at last, the data assimilation process for the estimation of the material parameters (section 2.3). In section 3, the sensitivity of the data assimilation parameters (section 3.1) and boundary conditions (section 3.2) and baseline stress state (section 3.3) for the mechanical problems are studied to assess their impact on the data assimilation outcomes. Hence, the mechanical characterization is performed for four *in-vivo* atherosclerotic lesions to analyze the performance of the method in real case scenarios (section 3.4). Insights, strengths and weaknesses of the methodology are then discussed in section 4 and final remarks are outlined in section 5.

## 2. Methods

**Figure 1:**
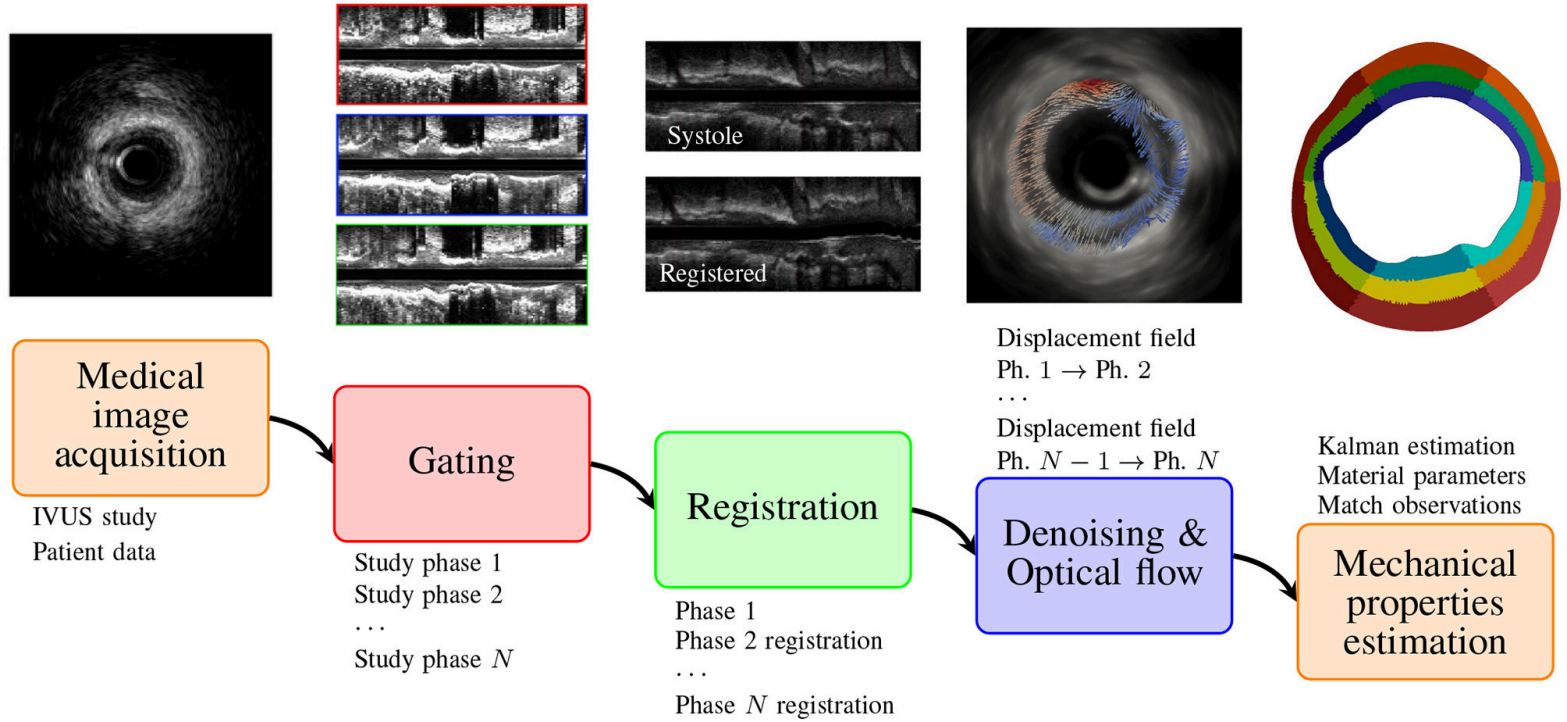
Proposed pipeline to estimate the patient-specific mechanical properties from IVUS medical images.

This section is divided in four parts. First, the IVUS imaging processing methods are described, where we present the procedures to obtain the displacement field of a specific vessel cross-section along the cardiac cycle (see Figure 1). Second, the mathematical model for the arterial mechanics is formulated, defining the mechanical equilibrium and the material constitutive behavior. Third, the data assimilation algorithm is presented as a tool to estimate unknown material properties in the mechanical models using the displacement field retrieved from the IVUS images. Finally, an efficient three-level parallelization scheme is described for high performance computing environments.

### 2.1. Image processing

The goal of the image processing stage is to deliver the displacement field of the vessel wall along the cardiac cycle at a particular site of interest within the artery. As this new methodology is a proof of concept, the data from the *in-vivo* cases will be extracted from a standard IVUS pullback as a retrospective study. As the transducer is axially displaced from frame to frame, only images corresponding to a single cardiac cycle can be extracted for each cross-section to obtain small topological variations between the images (spatial consistency). Hence, the extraction of the frames at a particular location is hindered due to the motion of the IVUS transducer exerted by the myocardium contraction. To overcome this issue, gating and registration procedures are performed using methods previously presented in Maso Talou et al. (2015, 2017). To retrieve the displacement field, a modified optical flow method is applied to the extracted frames at the site of interest. As follows, the treatment given to the IVUS images is briefly described.

#### 2.1.1. Gating

The gating method aims to recover the cardiac phase at each cross-sectional image of the study. To achieve this, a signal that measures the total motion of each frame is generated as

(1) $$\begin{array}{l} s(n)=\alpha_{g}\left[1-\frac{\sum_{i=1}^{H}\sum_{j=1}^{W}\left(I_{n}(i,j)-\mu_{n}\right)\left(I_{n+1}(i,j)-\mu_{n+1}\right)}{\sigma_{n}\sigma_{n+1}}\right]\\ +\;(1-\alpha_{g})\sum_{i=1}^{H}\sum_{j=1}^{W}-\left|\nabla I_{n}(i,j)\right|, \end{array}$$

where *I*_*n*_ is the *n*-th image of the study with a resolution of *H* × *W* pixels, μ_*n*_ and σ_*n*_ are the mean and standard deviation of the intensity at *I*_*n*_ and α_*g*_ a mixture parameter. The principal frequency mode of the signal *s*(*n*) at the physiological heart-frequency range (i.e., between 0.75 and 1.66 Hz) is extracted to obtain the mean cardiac frequency of the study, *f*_*m*_. Then, a low frequency signal *s*_*l*_(*n*) is generated by low-pass filtering *s*(*n*) with cut-frequency *f*_*c*_ = 1.4*f*_*m*_. If there is not severe arrhythmia during the IVUS acquisition, *s*_*l*_ presents one minimum per cardiac cycle related to the end-diastolic phase, thus, all frames for this phase are easily and directly extracted. Due to heartbeat period variability along the study, some of these minima can be displaced between *s* and *s*_*l*_, because of the lack of high frequencies contributions. To avoid such inconsistencies, we iteratively modify $f_{c}^{k}=\left(k+0.4\right)f_{m}$ (*k* is the current iteration number), recompute $s_{l}^{k}$ with the new cut-frequency $f_{c}^{k}$ and adjust each minimum of iteration *k*−1 to its nearest local minimum in $s_{l}^{k}$. Interestingly, the iterative scheme aids in cases with mild arrhythmia, i.e., where only few heartbeats of the study (not contiguous) present delay or omission of the P-wave. In those cases, the adjustment of the minima identified correctly the P-waves or collapsed the two minima to the same time position (this is the case when a P-wave did not occur and the heartbeat elapsed twice its period). In both of the previous cases, the minima are correct. In cases with severe arrhythmia, it is recommended the use of ECG signal and manual segmentation of minima for a proper gating. In the so-obtained phase, the cardiac contraction is at its minimum, and so, it corresponds to the beginning of the cardiac cycle, more precisely the beginning of the cardiac P-wave.

Since the heart frequency changes along the study, the heartbeats are sampled with a variable amount of frames. This variability in the heartbeat frequency affects mainly the relaxation process of the heart and, consequently, the length of the T-P interval. Despite this, the P-T interval remains almost invariant. Taking this fact into consideration, the end-diastolic instant for each cardiac cycle will be regarded as a reference for the definition of S cardiac phases. Each available frame of the P-T interval is then associated to a specific cardiac phase, obtaining phase-coherent volume datasets. Further details of the gating method, setup of the mixture parameter α_*g*_ and validation with *in-vivo* studies are described in Maso Talou et al. (2015).

#### 2.1.2. Registration

All phase-coherent volumes are registered (axially and transversally) against the volume dataset corresponding to the end-diastolic phase. This procedure is performed for each phase-coherent volume. The transversal registration is achieved by finding the in-plane rigid motion for each image in the current phase that best matches the frame image in the end-diastolic phase. To quantify the matching between two images, we use a maximum likelihood estimator presented in Cohen and Dinstein (2002) and Wachinger et al. (2008),

(2) $$c(I_{n},I_{m})=\sum_{i=1}^{H}\sum_{j=1}^{W}[I_{n}(i,j)-I_{m}(i,j)-\log(e^{2(I_{n}(i,j)-I_{m}(i,j))}+1)].$$

The rigid motion Ξ_*n*_ for each cross-section is then estimated by solving the following optimization problem

(3) $$\begin{array}{c} \Xi_{n}=\underset{\Xi^{*}}{\text{arg max }}c\left(I_{n}^{\text{D}},I_{n}^{s}(x(\Xi^{*}),y(\Xi^{*}))\right), \end{array}$$

where $I_{n}^{s}$ is the *n*-th cross-section of the phase-coherent volume corresponding to the *s*-th phase, *D* denotes the end-diastolic phase, and *I*(*x*(Ξ^*^), *y*(Ξ^*^)) is the image *I* after applying the rigid transformation defined by Ξ^*^ which is composed by an in-plane translation plus a rotation with respect to the image center.

By virtue of the myocardium contraction, the same cross-sections site at the different phases may be longitudinally displaced. Therefore, it is necessary to perform an axial registration to find the corresponding frames at different phases for the same transversal site. Thus, after transversal registration of all phase-coherent volumes, an axial registration against the end-diastolic phase is applied. For each frame of each phase-coherent volume (now transversally registered), the best matching frame in the end-diastolic volume is sought out. To diminish the computational burden, the search is limited to the 14 adjacent frames in the end-diastolic volume which is within the range of axial displacements of a transducer during the IVUS study (Arbab-Zadeh et al., 1999). To quantify the matching between two images, we use a neighborhood likelihood estimator defined as

(4) $$\begin{array}{l} c_{w}\left(I_{n}^{s},I_{m}^{\text{D}}\right)=\frac{\overset{w}{\underset{d=-w}{\sum }}\phi_{\sigma_{\text{G}}}\left(d\right)c\left(I_{n+d}^{s},I_{m+d}^{\text{D}}\right)}{\overset{w}{\underset{d=-w}{\sum }}\phi_{\sigma_{\text{G}}}\left(d\right)}, \end{array}$$

where ϕ_σ_G__ is a Gaussian weight function with σ_G_ standard deviation and *w* is the amount of adjacent frames used to establish the matching between the two sites centered at $I_{n}^{s}$ and $I_{m}^{\text{D}}$ respectively. It is important to note that *w* is not the search range fixed at 14 frames, but is the size of the neighborhood used for each comparison between two frames. Then, the position for axial registration, i. e., frame of the end-diastolic phase that best matches the current frame $I_{n}^{s}$ is given by

(5) $$\begin{array}{l} m=\underset{k=n-7,\ldots ,n+7}{\text{arg max}}c_{w}\left(I_{n}^{s},I_{k}^{\text{D}}\right). \end{array}$$

Finally, given the site of interest at the *n*-th frame of the end-diastolic phase volume, the set of frames that constitutes a sequence along the cardiac cycle at this site is $I=\left\{{Ĩ}_{n}^{s},s=1,\ldots ,S\right\}$, where ${Ĩ}_{n}^{s}$ is the *n*-th frame of the phase-coherent volume corresponding to phase *s* after transversal and axial registration. The reader is directed to Maso Talou et al. (2017) and Maso Talou (2017) for further details of the registration methods.

#### 2.1.3. Optical flow

For a pair (or sequence) of images, optical flow techniques aim at determining the displacement vector field that relates the points of both images (Horn and Schunck, 1981). Because optical flow strategies rely on the gray constancy assumption, a denoising procedure is performed over the sequence. The applied denoising method is a variation of the TV-L1 method (Rudin et al., 1992; Chan et al., 1999) which modifies the data term (absolute difference measurement of the images) by the negative maximum likelihood estimator assuming one image with gamma distributed noise and another noiseless image. Thus, the denoised image *I* corresponding to the noisy image *J* is obtained as

(6) $$I=\underset{Ĩ}{\text{arg min}}\int_{\Omega }[-\gamma_{d}\nu_{d}(J-Ĩ)+\delta_{d}^{-\gamma_{d}}e^{\gamma_{d}(J-Ĩ)}+\alpha_{d}|\nabla Ĩ|]d\Omega .$$

where Ω is the image domain, γ_*d*_, ν_*d*_, δ_*d*_ are parameters of the generalized gamma distribution that models the noise and α_*d*_ the regularization parameter for denoising.

Then, the optical flow is estimated for the denoised sequence of images using the method proposed in Brox et al. (2004). Particularly, the flow (i.e., the displacement field) is computed between the end-diastolic frame of the sequence to the other frames, corresponding to the different cardiac phases. Thus, the displacement field **u**^OF^ = (*u*^OF^, υ^OF^) between the end-diastolic frame *I*^D^ and the *s*-phase frame *I*^*s*^ is given by

(7) $$\begin{array}{l} {\text{u}}^{\text{OF}}=\overset{R}{\underset{r=1}{\sum }}\delta {\text{u}}^{r}, \end{array}$$

where δ**u**^*r*^ is the flow component corresponding to the image resolution *r* that is obtained as

(8) $$\begin{array}{l} \delta {\text{u}}^{r}=\underset{\delta \text{u}}{\text{arg min}}\underset{\Omega }{\underset{\;}{\int }}[\psi \left({\left\|\frac{\partial I^{r}}{\partial t}+\nabla I^{r}\cdot \delta \text{u}\right\|}_{G_{\rho }}^{2}\right)\\ \;+\;\alpha_{o}\psi {(\left\|\nabla ({\text{u}}^{r-1}+\delta \text{u})\right\|}_{F}^{2}]d\Omega , \end{array}$$

where ${\text{u}}^{r-1}=\overset{r-1}{\underset{t=1}{\sum }}\delta {\text{u}}^{t}$, ∥·∥_*F*_ is the Frobenius norm, α_*o*_ is the regularization parameter for optical flow. The function ψ and the weighted norm ∥·∥_*G*_ρ__ are defined by

$$\begin{array}{l} \psi (x)=2\kappa^{2}\sqrt{1+\frac{x}{\kappa^{2}}},\\ {\left\|\frac{\partial I^{r}}{\partial t}+\nabla I^{r}\cdot \delta \text{u}\right\|}_{G_{\rho }}^{2}=G_{\rho }*{\left(\frac{\partial I^{r}}{\partial x}\right)}^{2}\delta u^{2}+G_{\rho }*{\left(\frac{\partial I^{r}}{\partial y}\right)}^{2}\delta \upsilon^{2}\\ \;+G_{\rho }*{\left(\frac{\partial I^{r}}{\partial t}\right)}^{2}+2G_{\rho }*\left(\frac{\partial I^{r}}{\partial x}\frac{\partial I^{r}}{\partial y}\right)\delta u\delta \upsilon \\ \;+2G_{\rho }*\left(\frac{\partial I^{r}}{\partial x}\frac{\partial I^{r}}{\partial t}\right)\delta u+2G_{\rho }*\left(\frac{\partial I^{r}}{\partial y}\frac{\partial I^{r}}{\partial t}\right)\delta \upsilon \end{array}$$

where *G*_ρ_ is the Gaussian kernel with ρ standard deviation and ^*^ is the convolution operator. Note that the flow **u**^OF^ is the displacement field between *I*^D^ and *I*^*s*^, then the temporal derivative is estimated as the variation of the intensity between such frames.

Such strategy defines all displacement fields along the cardiac sequence at the same reference phase (the end-diastolic phase), which eases the integrability of the data into the assimilation process introduced in section 2.3.

#### 2.1.4. Patient-specific geometric model

**Figure 2:**
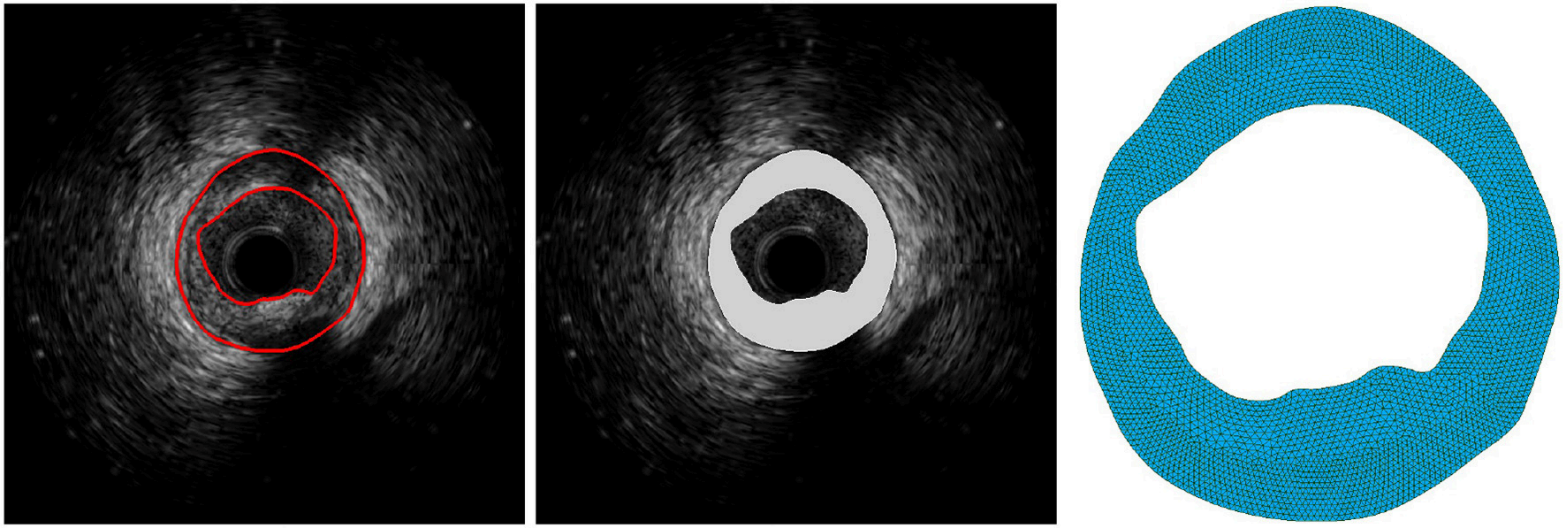
Vessel segmentation and geometric model generation: **(Left)** segmentation of the intima-media area over the IVUS image; **(Middle)** extruded volume of the intima-media area; **(Right)** final 3D mesh.

Using an IVUS study gated at the end-diastolic phase, a geometrical model for a frame of interest is constructed (see Figure 2). First, the intima-media area is manually segmented by a specialist from the image using cubic splines to obtain a 2D patient-specific geometry. Then, the 2D geometry is extruded 0.05 mm in the axial direction to render a 3D slice of the arterial vessel. The mesh generation from this geometry is described later in section 2.2.5 when the numerical scheme for the mechanical problem is introduced.

### 2.2. Mechanical setup for the arterial wall

In this section, the main ingredients from continuum mechanics required to describe the mathematical models are briefly summarized. For further details the reader may refer to Ares (2016) and Blanco et al. (2016).

**Figure 3:**
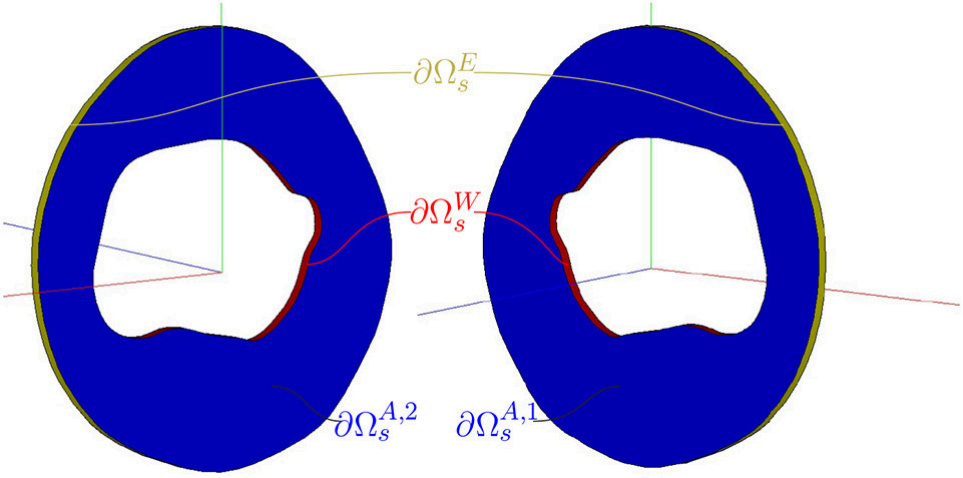
Cross-sectional slice of an arterial vessel. Description of the spatial domain Ω_*s*_ and boundaries $\partial \Omega_{s}=\partial \Omega_{s}^{W}\cup \partial \Omega_{s}^{E}\cup \partial \Omega_{s}^{A}$ of the arterial wall.

Let us consider the domain of a cross-sectional slice of the vessel wall. Its *spatial* configuration in the Euclidean space is denoted by Ω_*s*_, with boundary $\partial \Omega_{s}=\partial \Omega_{s}^{W}\cup \partial \Omega_{s}^{E}\cup \partial \Omega_{s}^{A}$, where $\partial \Omega_{s}^{W}$ represents the interface between the vessel and the blood, $\partial \Omega_{s}^{E}$ the external surface, and $\partial \Omega_{s}^{A}={⋃}_{i=1}^{2}\partial \Omega_{s}^{A,i}$ stands for the set of 2 cross-sectional (non-physical) axial boundaries for the vessel slice (see Figure 3). The unit outward normal vector is denoted by **n**_*s*_. The coordinates at this configuration are denoted by **x**_*s*_. A *material* configuration, used as a reference configuration, is denoted by Ω_*m*_, with coordinates **x**_*m*_. In the present context, Ω_*s*_ stands for the configuration at which mechanical equilibrium is achieved for a given load condition (diastolic, systolic or any other loaded state of the arterial wall). Residual stresses are neglected, therefore, the material configuration Ω_*m*_ is both load-free and stress-free.

The displacement field mapping points from the material into the spatial configuration is denoted by **u**. Then, we characterize the deformation mapping from Ω_*m*_ onto Ω_*s*_ and its inverse by the following expressions,

(9) $$\begin{array}{l} {\text{x}}_{s}=\chi_{m}\left({\text{x}}_{m}\right)={\text{x}}_{m}+{\text{u}}_{m}, \end{array}$$

(10) $$\begin{array}{l} {\text{x}}_{m}=\chi_{s}\left({\text{x}}_{s}\right)=\chi_{m}^{-1}\left({\text{x}}_{s}\right)={\text{x}}_{s}-{\text{u}}_{s}, \end{array}$$

where subscripts *m* and *s* denote the descriptions of the fields in the material and spatial configurations, respectively. Thus, the displacement vector field is given by

(11) $$\begin{array}{l} {\text{u}}_{s}\left({\text{x}}_{s}\right)={\left({\text{u}}_{m}\left({\text{x}}_{m}\right)\right)}_{s}={\text{u}}_{m}\left(\chi_{m}^{-1}\left({\text{x}}_{s}\right)\right), \end{array}$$

and its gradients with respect to material and spatial coordinates are, respectively, obtained as

(12) $$\begin{array}{l} {\text{F}}_{m}=\nabla_{m}\chi_{m}=\text{I}+\nabla_{m}{\text{u}}_{m}, \end{array}$$

(13) $$\begin{array}{l} {\text{f}}_{s}=\nabla_{s}\chi_{s}=\nabla_{s}\chi_{m}^{-1}=\text{I}-\nabla_{s}{\text{u}}_{s}. \end{array}$$

Observe that ${\left[{\text{F}}_{m}^{-1}\right]}_{s}={\text{f}}_{s}$ and ${\left[{\text{f}}_{s}^{-1}\right]}_{m}={\text{F}}_{m}$. Arterial wall tissues are assumed to behave as incompressible materials, which is mathematically represented by the following kinematic condition

(14) $$\begin{array}{l} \det{\text{F}}_{m}=1. \end{array}$$

In a general case the load state of the model of an arterial cross-section is characterized as follows. Neumann boundary conditions are considered to be given by the forces exerted by the blood flow over $\partial \Omega_{s}^{W}$, i.e., through a traction field ${\text{t}}_{s}^{W}$ which is considered to be characterized as ${\text{t}}_{s}^{W}=p_{s}{\text{n}}_{s}$ (here we only consider the pressure load, and neglect the shear forces imprinted by the blood flow on the vessel wall), and by the tethering tractions ${\text{t}}_{s}^{A,i}$ acting over $\partial \Omega_{s}^{A,i}$, *i* = 1, 2. For ease of notation, the tethering tractions are grouped into ${\text{t}}_{s}^{A}$, which is defined over the whole $\partial \Omega_{s}^{A}$. The action of the surrounding tissues is introduced as an elastic traction over the external boundary, which is characterized by the elastic parameter τ and depends on the displacement field at this boundary (see further details in section 2.2.1). These tractions, representing the external tissues influence, only act over the external surface $\partial \Omega_{s}^{E}$ in the physiological pressure range, i.e., at end-diastolic pressure or higher. That is, during the preload problem (see section 2.2.2), the boundary ∂Ω_*E*_ is an homogeneous Neumann boundary (except for a small region of arc length Δ = 0.1mm which is fixed to remove rigid motions).

The mechanical problem in variational form is framed as a saddle-point problem to accommodate the incompressibility constraint through the corresponding Lagrange multiplier, i.e., the pressure field in the solid domain.

Next, two variational formulations are presented which formalize the concept of mechanical equilibrium for the so-called preload and forward problems. In the *preload problem*, the known configuration is that one at which the body is at equilibrium (the spatial domain), and the unknown configuration is the material configuration used to define the constitutive equations. In the *forward problem*, the known configuration is the material one, while the unknown configuration is the one where equilibrium actually occurs.

#### 2.2.1. Forward problem

When the material (load- and stress-free) configuration Ω_*m*_ is known, the variational Equation (16) can be cast in the material domain, yielding what we define as the *forward problem*. The variational formulation then reads: given the material description of the loads, *p*_*m*_ and ${\text{t}}_{m}^{A,i}$, find (**u**_*m*_, λ_*m*_) in $U_{m}\times L_{m}$ such that

(15) $$\begin{array}{l} \underset{\Omega_{m}}{\int }\left(1-\det{\text{F}}_{m}\right){\hat{\lambda }}_{m}d\Omega_{m}-\underset{\Omega_{m}}{\int }\lambda_{m}\left({\text{F}}_{m}^{-T}\cdot \nabla_{m}{\hat{\text{u}}}_{m}\right)\det{\text{F}}_{m}d\Omega_{m}\\ +\underset{\Omega_{m}}{\int }\left({\text{S}}_{m}\left({\text{E}}_{m}\right)\right)\cdot \overset{\cdot }{\text{E}}\left({\hat{\text{u}}}_{m}\right)d\Omega_{m}\\ =\underset{\partial \Omega_{m}^{E}}{\int }\tau \left({\text{u}}_{m}-{\left({\text{u}}_{d}+{\text{u}}^{\text{OF}}\right)}_{m}\right)\cdot {\hat{\text{u}}}_{m}|{\text{F}}_{m}^{-T}{\text{n}}_{0}^{E}|\det{\text{F}}_{m}d\partial \Omega_{m}^{E}\\ +\underset{\partial \Omega_{m}^{W}}{\int }\left(p_{m}{\text{F}}_{m}^{-T}{\text{n}}_{0}^{W}\cdot {\hat{\text{u}}}_{m}\right)\det{\text{F}}_{m}d\partial \Omega_{m}^{W}\\ +\overset{2}{\underset{i=1}{\sum }}\underset{\partial \Omega_{m}^{A,i}}{\int }\left({\text{t}}_{m}^{A,i}\cdot {\hat{\text{u}}}_{m}\right)|{\text{F}}_{m}^{-T}{\text{n}}_{0}^{A,i}|\det{\text{F}}_{m}d\partial \Omega_{m}^{A,i}\\ \;\forall \left({\hat{\text{u}}}_{m},{\hat{\lambda }}_{m}\right)\in V_{m}\times L_{m}, \end{array}$$

where $\overset{\cdot }{\text{E}}\left({\hat{\text{u}}}_{m}\right)=\frac{1}{2}\left[{\text{F}}_{m}^{T}\left(\nabla_{m}{\hat{\text{u}}}_{m}\right)+{\left(\nabla_{m}{\hat{\text{u}}}_{m}\right)}^{T}{\text{F}}_{m}\right]$, **n**_0_ is the unit outward normal vector in the material configuration. Recall that τ is the elastic parameter characterizing the response of the surrounding media, **u**^OF^ is the displacement field which maps the end-diastolic to the spatial configuration where equilibrium is achieved (see Equation 7), and **u**_*d*_ is the displacement field which maps points from the material to the end-diastolic configuration. Also, $U_{m}$, $V_{m}$, and $L_{m}$ are the counterparts of $U_{s}$, $V_{s}$, and $L_{s}$, respectively, with functions defined in Ω_*m*_.

Acceleration terms have also been neglected, since the stresses associated to such inertial forces is much smaller than those of constitutive origin (Ares, 2016; Blanco et al., 2016).

#### 2.2.2. Preload problem

Given the equilibrium configuration Ω_*s*_, the variational formulation reads: given the loads ${\text{t}}_{s}^{W,n}$ and ${\text{t}}_{s}^{A}$, find $\left({\text{u}}_{s},\lambda_{s}\right)\in U_{s}\times L_{s}$ such that

(16) $$\begin{array}{l} \underset{\Omega_{s}}{\int }\left[-\lambda_{s}\mathrm{div}{\hat{\text{u}}}_{s}+\sigma_{s}\cdot \varepsilon_{s}\left({\hat{\text{u}}}_{s}\right)\right]d\Omega_{s}-\underset{\Omega_{s}}{\int }\left[1-\det{\text{F}}_{s}^{-1}\right]{\hat{\lambda }}_{s}d\Omega_{s}=\\ \underset{\partial \Omega_{s}^{W}}{\int }t_{s}^{W,n}{\text{n}}_{s}\cdot {\hat{\text{u}}}_{s}d\partial \Omega_{s}^{W}+\overset{2}{\underset{i=1}{\sum }}\underset{\partial \Omega_{s}^{A,i}}{\int }{\text{t}}_{s}^{A,i}\cdot {\hat{\text{u}}}_{s}d\partial \Omega_{s}^{A,i}\\ \forall \left({\hat{\text{u}}}_{s},{\hat{\lambda }}_{s}\right)\in V_{s}\times L_{s}, \end{array}$$

where $\varepsilon_{s}\left(\hat{\text{u}}\right)=\frac{1}{2}\left(\nabla_{s}\hat{\text{u}}+\nabla_{s}{\hat{\text{u}}}^{T}\right)$ is the strain rate tensor, $L_{s}=L^{2}\left(\Omega_{s}\right)$ and $U_{s}=\left\{{\text{u}}_{s}\in {\text{H}}^{1}\left(\Omega_{s}\right),{\text{u}}_{s}\text{satisfies essential b.c.}\right\}$ are, respectively, the linear space for pressures and the linear manifold for kinematically admissible displacements, and $V_{s}={\hat{\text{u}}}_{s}\in {\text{H}}^{1}\left(\Omega_{s}\right),{\hat{\text{u}}}_{s}$ satisfies homogeneous essential b.c.} is the space of kinematically admissible variations. Also, **σ**_*s*_ is related to the second Piola-Kirchhoff stress tensor **S**_*m*_ through

(17) $$\begin{array}{l} \sigma_{s}=\frac{1}{\det{\text{F}}_{s}}{\text{F}}_{s}{\left({\text{S}}_{m}\left({\text{E}}_{m}\right)\right)}_{s}{\text{F}}_{s}^{T}. \end{array}$$

where **S**_*m*_ is a function of the Green-Lagrange deformation tensor ${\text{E}}_{m}=\frac{1}{2}\left({\text{F}}_{m}^{T}{\text{F}}_{m}-\text{I}\right)$ via a constitutive equation (see section 2.2.4).

In this work the preload problem is used to obtain the material configuration that enables an appropriate calculation of the stress field, which realizes the equilibrium in the end-diastolic configuration. Note that in this case the action of the surrounding media is omitted. This is due to the fact that our hypothesis considers the end-diastolic configuration as a reference configuration for the elastic response of the external tissues.

#### 2.2.3. Equilibrium problems for a given set of material parameters

The *preload problem* is a mandatory step toward characterizing the mechanical state (the stress state) of the arterial wall in a geometry obtained from medical images (e.g., the end-diastolic geometry) with given baseline hemodynamics loads. In this context, these loads are given by the end-diastolic pressure, also called preload pressure, and by the axial stretch caused by tethering forces. The material configuration is required because it is used to define constitutive equations, without which the forward problem cannot properly be formulated. In our case, such baseline geometry is obtained from IVUS study, while the baseline hemodynamics loads (the blood pressure) are estimated from patient specific data. Just after solving the preload problem, the baseline mechanical state, that is the stress state due to the preload pressure (i.e., pressure at diastole), is adequately determined and the displacement field **u**_*s*_—that maps the material (load-free) configuration to the diastolic configuration– is recalled as **u**_*d*_. Then, the *forward problem* is solved to determine the equilibrium configuration for other hemodynamics loads occurring during the cardiac cycle. In that manner both problems are synergically coupled to solve a forward problem from an adequately preloaded configuration.

In practice, a set of physiological loads for the vessel will be given. Individualizing the diastolic pressure level as *p*_*s*_, a set of pressure loads between diastole and systole can be listed as {*p*_*s*_1__, …, *p*_*s*_*S*__}. Through the forward problem, each load *p*_*s*_*i*__ will be in correspondence with an unknown spatial configuration Ω_*s*_*i*__, *i* = 1, …, *S*. Notice then that, for a given set of material parameters, the preload problem is solved only once and so the *forward problem* is solved for each load *p*_*s*_*i*__ in the set of physiological loads.

#### 2.2.4. Constitutive models

The main components of the atherosclerotic plaque, i.e., fibrotic, lipidic and calcified tissues, are modeled as isotropic Neo-Hookean materials. In Walsh et al. (2014), it is shown that fibrotic tissue in illiac plaque presents a quasi isotropic behavior. Different from the fibrotic tissue, the lipidic and calcified tissues do not display any contribution of smooth muscle cells or oriented fibers that may endow their structures with anisotropic behavior, what suggests that an isotropic hypothesis for these materials is reasonable.

The isotropic Neo-Hookean model is suitable for materials under large deformations where the stress-strain relationship behaves as non-linear, elastic, isotropic and independent of strain rate. Also, the model assumes an ideal elastic material at every strain level which, for physiological ranges, is satisfied by many biological tissues. The stress-strain relationship for a Neo-Hookean material derives from the strain energy function

(18) $$\begin{array}{l} \psi =\frac{c}{2}\left({\bar{I}}_{1}-3\right), \end{array}$$

where *c* is the material parameter that characterizes the stiffness of the material and ${\bar{I}}_{1}$ is the first isochoric invariant of the Cauchy-Green tensor

(19) $$\begin{array}{l} {\bar{I}}_{1}=\mathrm{Tr}{(\text{C}}_{m}{\left(\det{\text{F}}_{m}\right)}^{-2/3}), \end{array}$$

with ${\text{C}}_{m}={\text{F}}_{m}^{T}{\text{F}}_{m}$. Then, the second Piola-Kirchhoff stress tensor (and the **σ**_*m*_ through Equation 17) is obtained as

(20) $$\begin{array}{l} {\text{S}}_{m}\left({\text{E}}_{m}\right)=\frac{\partial \psi }{\partial {\text{E}}_{m}}. \end{array}$$

#### 2.2.5. Numerical methods

The preload and forward problems are linearized using the Newton-Raphson method. Linear tetrahedral finite elements for both displacement and pressure fields are used for the spatial discretization of the corresponding linearized problems. To stabilize the problem in the sense of the inf-sup condition, the linearized (forward and preload) problems are modified adding a diffusive term in the pressure equation. For the analysis of the proposed approach, four patient-specific 3D geometries were obtained using the technique described in section 2.1.4. These geometries were discretized using Netgen 3D using a characteristic element size ranging from 10μm to 40μm, resulting in meshes with 6,521, 7,516, 4,835, and 3,808 nodes for the cases 1–4, respectively. All these steps are performed using an in-house solver. The resulting systems of linear equations are solved using a direct solver based on LU factorization from the SuperLU library (Li and Demmel, 2003). Further details regarding the linearization and numerical schemes can be found in Ares (2016) and Blanco et al. (2016).

The Newton iterative scheme in both equilibrium problems finishes when $∥{\text{u}}_{s}^{m+1}-{\text{u}}_{s}^{m}{∥}_{L^{\infty }}<10^{-4}$ mm and $∥\lambda_{s}^{n+1}-\lambda_{s}^{n}{∥}_{L^{\infty }}\;<\;1$ Pa. Such convergence criterion was chosen to yield a higher precision than the optical flow processing applied to IVUS images (16·10^−3^ mm assuming pixel precision).

### 2.3. Data assimilation

In the data assimilation process, the displacement field **u**^OF^ obtained using the optical flow technique as explained in section 2.1 and the mechanical models presented in the previous section (section 2.2) are integrated by an unscented Kalman filter. Let us define a partition for the domain of analysis $\Omega_{s}={⋃}_{j=1}^{M}\Omega_{s}^{j}$ composed by *M* disjoint regions. Each region $\Omega_{s}^{i}$ is characterized by its own material parameter, say *c*_*i*_, see Equation (18). The axial loads ${\text{t}}_{s_{i}}^{A,n}$, the pressure level *p*_*s*_*i*__ and the displacement fields ${\text{u}}_{s_{i}}^{\text{OF}}$ (obtained by optical flow techniques) are known at *S* cardiac phases (*i* = 1, …, *S*). Since our mechanical problem is time-independent, the time instants in the context of the Kalman filter simply correspond to filter iterations, while at each iteration all forward problems must be solved. By using the mechanical constitutive models, the material parameters grouped as θ = (*c*_1_, …, *c*_*M*_), are estimated such that

(21) $$\begin{array}{l} \theta =\underset{\hat{\theta }}{\text{arg min}}\overset{S}{\underset{i=1}{\sum }}∥{\text{u}}_{s_{i}}^{\text{MO}}\left(\hat{\theta }\right)-{\text{u}}_{s_{i}}^{\text{OF}}{∥}_{L_{2}}^{2}, \end{array}$$

where ${\text{u}}_{s_{i}}^{\text{MO}}\left(\hat{\theta }\right)$ is the displacement field at the configuration *s*_*i*_ obtained by solving the preload and forward problems (described in section 2.2) with pressure level *p*_*s*_*i*__ and material parameters $\hat{\theta }$.

The solution of the parameter identification problem eqution (21), satisfies the discrete dynamic nonlinear system presented as follows

(22) $$\begin{array}{l} \begin{array}{cc} X_{k}^{a} & =f\left(X_{k-1}^{a},t_{k-1}\right)+W_{k},\\ Z_{k} & =h\left(X_{k}^{a},t_{k}\right)+V_{k}, \end{array} \end{array}$$

where $X_{k}^{a}$ is the augmented state vector

(23) $$\begin{array}{l} X_{k}^{a}={\left[{\text{u}}_{s_{1}}^{k}\left(\text{x}\right),\ldots ,{\text{u}}_{s_{S}}^{k}\left(\text{x}\right),\lambda_{s_{1}}^{k}\left(\text{x}\right),\ldots ,\lambda_{s_{S}}^{k}\left(\text{x}\right),c_{1},\ldots ,c_{M}\right]}^{T}, \end{array}$$

which contains the displacement **u**_*s*_*i*__ and pressure λ_*s*_*i*__ fields for all forward problems *i* = 1, …, *S*, and the material parameters of all regions of the domain θ = (*c*_1_, …, *c*_*M*_); $f\left(X_{k}^{a},t_{k}\right)$ is the operator that sequentially solves the preload and all forward problems for parameters and initial state conditions in $X_{k}^{a}$ at filter iteration *t*_*k*_ (recall that these problems are time-independent, and so the dependence on time is ruled out in practice); *W*_*k*_ are the model errors at the *k*-th step; $h\left(X_{k}^{a},t_{k}\right)=\text{H}X_{k}^{a}$ is a linear observation operator represented by the block matrix

(24) $$\text{H}=[\begin{array}{ccc} I_{uu} & 0_{u\lambda } & 0_{u\theta } \end{array}],$$

where block matrix indexes indicate the corresponding dimensions; *Z* is the set of optical flow observations at each cardiac phase, described by the column vector

(25) $$\begin{array}{l} Z={\left[{\text{u}}_{s_{1}}^{\text{OF}}\left(\text{x}\right),\ldots ,{\text{u}}_{s_{S}}^{\text{OF}}\left(\text{x}\right)\right]}^{T}, \end{array}$$

where ${\text{u}}_{s_{i}}^{\text{OF}}\left(\text{x}\right)$ is the displacement field obtained by the optical flow technique for the cardiac phase *i*, *i* = 1, …, *S* (observe that for the present case of static problems, the observations are fixed concerning the dynamics of the data assimilation process); *V* is the vector of optical flow and interpolation errors for the observation vector *Z*.

To obtain an estimate of the parameters θ, a reduced ordered unscented Kalman filter (ROUKF) (Julier and Uhlmann, 2002, 2004) is applied to the system described in Equation (22). The filter comprises the following steps

1. Spherical sigma-points generation $\sigma_{i}^{\left(n\right)},i=1,\ldots ,M+1$ with their corresponding weights *w*^(*i*)^ (see Julier, 2003) and initialization of the variables
  (26) $$\begin{array}{l} R_{0}=\sigma_{\text{OF}}I_{uu};\;L_{0}=\left[\begin{array}{c} L_{0}^{X}\\ L_{0}^{\theta } \end{array}\right]=\left[\begin{array}{c} L_{0}^{u}\\ L_{0}^{\lambda }\\ L_{0}^{\theta } \end{array}\right]=\left[\begin{array}{c} 0_{u\theta }\\ 0_{\lambda \theta }\\ I_{\theta \theta } \end{array}\right];\\ \;U_{0}^{-1}=\left[\begin{array}{ccc} \sigma_{{\hat{c}}_{1}} & \ldots & 0\\ \vdots & \ddots & \vdots \\ 0 & \ldots & \sigma_{{\hat{c}}_{M}} \end{array}\right], \end{array}$$
  (27) $$\begin{array}{l} X_{0}^{a}={\left[{\hat{X}}_{0}^{+},{\hat{\theta }}_{0}^{+}\right]}^{T}={\left[0_{\text{u}},0_{\lambda },{\hat{\theta }}_{0}\right]}^{T}, \end{array}$$
  (28) $$\begin{array}{l} {\text{P}}_{0}^{+}={\text{L}}_{0}{\text{U}}_{0}^{-1}{\text{L}}_{0}^{T}, \end{array}$$
  where σ_OF_ is the uncertainty of the computed optical flow and σ_ĉ_*i*__ is the uncertainty of the parameter *c*_*i*_, *i* = 1, …, *M*. The sensitivity analysis of the uncertainty value is studied in section 3.1.
2. The *prediction* step
  (29) $$\begin{array}{l} \;{\hat{X}}_{k-1}^{\left(i\right)}={\hat{X}}_{k-1}^{+}+L_{k-1}^{X}\sqrt{U_{k-1}^{-1}}\sigma_{i}^{\left(n\right)},\;i=1,\ldots ,M+1,\\ \;{\hat{\theta }}_{k-1}^{\left(i\right)}={\hat{\theta }}_{k-1}^{+}+L_{k-1}^{\theta }\sqrt{U_{k-1}^{-1}}\sigma_{i}^{\left(n\right)},\;i=1,\ldots ,M+1,\\ \;[\begin{array}{c} \left({\hat{X}}_{k}^{\left(i\right)}\right)\\ \left({\hat{\theta }}_{k}^{\left(i\right)}\right) \end{array}]=f\left(\left[\begin{array}{c} \left({\hat{X}}_{k-1}^{\left(i\right)}\right)\\ \left({\hat{\theta }}_{k-1}^{\left(i\right)}\right) \end{array}\right],t_{k-1}\right),\\ \;{\hat{X}}_{k}^{-}=\sum_{i=1}^{M+1}w^{\left(i\right)}{\hat{X}}_{k}^{\left(i\right)},{\hat{\theta }}_{k}^{-}=\sum_{i=1}^{M+1}w^{\left(i\right)}{\hat{\theta }}_{k}^{\left(i\right)},{\hat{Z}}_{k}=\sum_{i=1}^{M+1}w^{\left(i\right)}{\hat{Z}}_{k}^{\left(i\right)}. \end{array}$$
3. The *correction* step
  (30) $$\begin{array}{l} \;L_{k}^{X}={\hat{X}}_{k}^{\left(*\right)}D_{w}{\left(\sigma^{\left(*\right)}\right)}^{T},\;L_{k}^{\theta }={\hat{\theta }}_{k}^{\left(*\right)}D_{w}{\left(\sigma^{\left(*\right)}\right)}^{T},\\ {\left\{HL\right\}}_{k}={\hat{Z}}_{k}^{\left(*\right)}D_{w}{\left(\sigma^{\left(*\right)}\right)}^{T},\\ \;P_{w}=\sigma^{\left(*\right)}D_{w}{\left(\sigma^{\left(*\right)}\right)}^{T},\\ \;U_{k}=P_{w}+{\left\{HL\right\}}_{k}^{T}R_{k}^{-1}{\left\{HL\right\}}_{k},\\ \;{\hat{X}}_{k}^{+}={\hat{X}}_{k}^{-}+L_{k}^{X}U_{k}^{-1}{\left\{HL\right\}}_{k}^{T}R_{k}^{-1}\left(Z-{\hat{Z}}_{k}\right),\\ \;{\hat{\theta }}_{k}^{+}={\hat{\theta }}_{k}^{-}+L_{k}^{\theta }U_{k}^{-1}{\left\{HL\right\}}_{k}^{T}R_{k}^{-1}\left(Z-{\hat{Z}}_{k}\right). \end{array}$$
  The matrices $\sigma^{\left(*\right)},{\hat{\text{X}}}_{k}^{\left(*\right)},{\hat{\text{Z}}}_{k}^{\left(*\right)},{\hat{\theta }}_{k}^{\left(*\right)}$ are the *M* × (*M*+1) matrices whose columns are the vectors $\sigma^{\left(i\right)},{\hat{X}}_{k}^{\left(i\right)},{Ẑ}_{k}^{\left(i\right)},{\hat{\theta }}_{k}^{\left(i\right)}$ with *i* = 1, …, *M*+1, respectively. **D**_*w*_ is the diagonal (*M*+1) × (*M*+1) matrix with values $D_{ii}=w^{\left(i\right)},i=1,\ldots ,M+1$, i.e., the sigma-point weights.
4. If stop criteria is not achieved, go to step 2 and *k* = *k*+1.

In this iterative scheme, the model errors *W*_*k*_ (inaccuracies in the solution of the preload and forward problems) have been neglected. The stop criteria used in this work is a fixed number of iterations that is reported for each study case in section 3.

In this work, *c* was reparametrized as $c=2^{\hat{\theta }}$ (this approach was introduced in Bertoglio et al., 2012) allowing $\hat{\theta }$ to vary in the whole ℝ (as occurs in the presented formulation 29, 30) without delivering invalid values for *c*.^1^

### 2.4. Parallelization scheme

**Figure 4:**
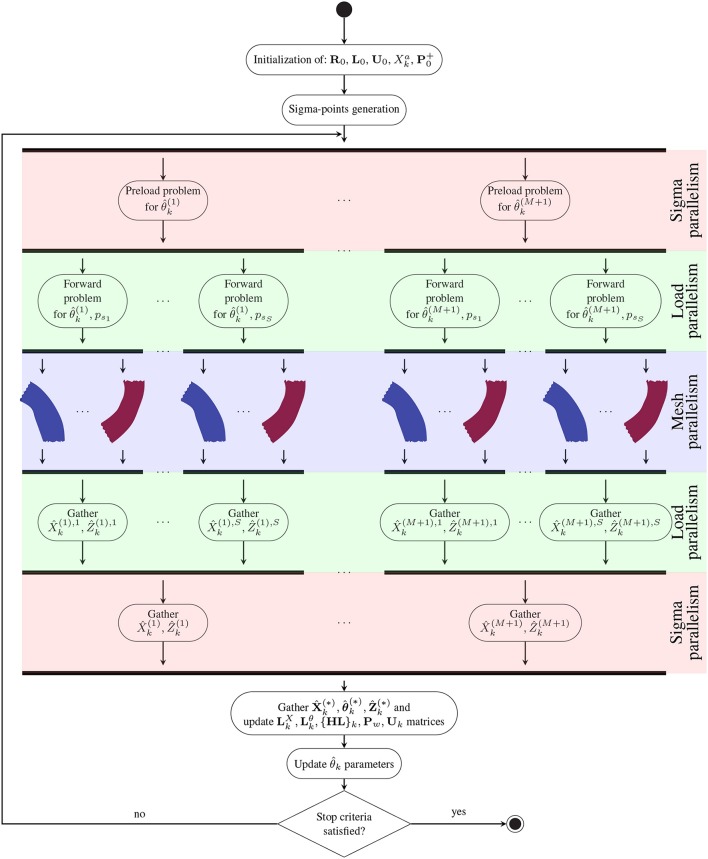
Activity diagram depicting the parallel workflow for data assimilation. The three levels of parallelism are highlighted on the right side: (i) Parallelization of the sigma points that are solved at the same time without communication among the threads; (ii) Parallelization of each load state of the artery (i.e., one load per cardiac phase) that is fully parallelized without communication among the threads; and (iii) Parallelization of the FEM problem by mesh partitioning.

The data assimilation scheme is a computationally demanding task. However, it presents many independent or low dependent tasks. Firstly, notice that all sigma point predictions can be computed in parallel. As the forward problem is static, all forward problems (one per load, for *S* different loads) are computed in parallel and an extended observation vector Ẑ^(*i*)^ = [Ẑ^(*i*), 1^, …, Ẑ^(*i*), *S*^]*T* for the *i*-th sigma-point is created by appending the predicted displacements Ẑ^(*i*), *j*^ of the *j*-th forward problem corresponding to the pressure load *p*_*s*_*j*__. In that manner, at each Kalman iteration, the observations of all frames, are processed at once. In turn, the forward problem itself is parallelized by partitioning the mesh and communicating among subdomains the results of the local operations in both assembling and solving stages. Partitioning is accomplished using ParMETIS (Karypis and Kumar, 1998), and the solution is achieved using the SuperLU library (Li and Demmel, 2003). Following such parallelization scheme, and assuming there are enough computational resources, the cost per iteration of the data assimilation process equals the cost of the computation of one preload problem plus one forward problem, regardless the number of cardiac phases or sigma-points employed (i.e., regardless the number of parameters to be estimated). Note that the cost of the Kalman filter increases as more parameters are estimated, although when compared to the computations required for solving the mechanical equilibrium problems this increment is insignificant (only a few dozens of parameters will be required in the worst case). In Figure 4, the activity diagram for the proposed parallel scheme is presented. Thus, the data assimilation process is HPC ready and, even, capable to handle large scale FEM problems.

## 3. Results

In what follows, sensitivity analyses are carried out to study the variation of the parameter estimation with respect to: the parameter uncertainties, boundary conditions and baseline stress state of the mechanical model (sections 3.1, 3.2, and 3.3, respectively). From these analyses, a reasonable setup of the data assimilation parameters and mechanical conditions is obtained for the present context of material identification in patient-specific models. Finally, in section 3.4, 4 patient-specific mechanical models are derived from *in-vivo* IVUS studies and the obtained displacement errors between the model predictions—with its parameters adjusted by data assimilation—and the optical flow observations are assessed.

### 3.1. Uncertainty parameters sensitivity

Let us define a homogeneous ring-shaped domain Ω_*s*_ with Neo-Hookean constitutive behavior (see Equation 18). The inner and outer radius of the ring are 2 and 2.71mm, respectively. The size and proportions are chosen to approximate an idealized coronary artery. Loads of *t*^*W, n*^ = 80 mmHg and *t*^*W, n*^ = 120 mmHg are applied over the inner surface for the preload and forward problems, respectively, and tethering tractions ${\text{t}}_{s}^{A,i}$ are considered such that an axial stretch of 10% is prescribed. At the outer surface, homogeneous Neumann boundary conditions are assumed (τ = 0 in Equation 16). To avoid rigid movements in this idealized geometry, only radial displacement is allowed for 4 equidistant nodes at the luminar perimeter. The forward operator *f*, which comprises the preload and forward problems (see Equations 15, 16), is solved at each filter iteration with an iterative scheme where a Newton-Raphson linearization procedure is applied as described in section 2.2.5 (further details in Blanco et al., 2016).

Using this setting, we create an *in-silico* experiment to analyze: (i) the sensitivity of the parameter estimates $\hat{\theta }$ with respect to the σ_*Z*_ (the observations uncertainty, previously referred to as σ_OF_); and (ii) the sensitivity of the parameter estimates $\hat{\theta }$ with respect to the σ_θ_ (the estimate uncertainty). Thus, the observations are generated by computing *Z* = *h*(*f*(*X*^*t*^)) where $X^{t}=\left[0_{\text{u}},0_{\lambda },\theta^{t}\right]$ is the true augmented state vector with the solution parameters *c*^*t*^ = 2^θ^*t*^^ for the experiment. In this particular case, the domain is homogeneous and the constitutive model has only one parameter (*c*), then, only one parameter is estimated.

**Figure 5:**
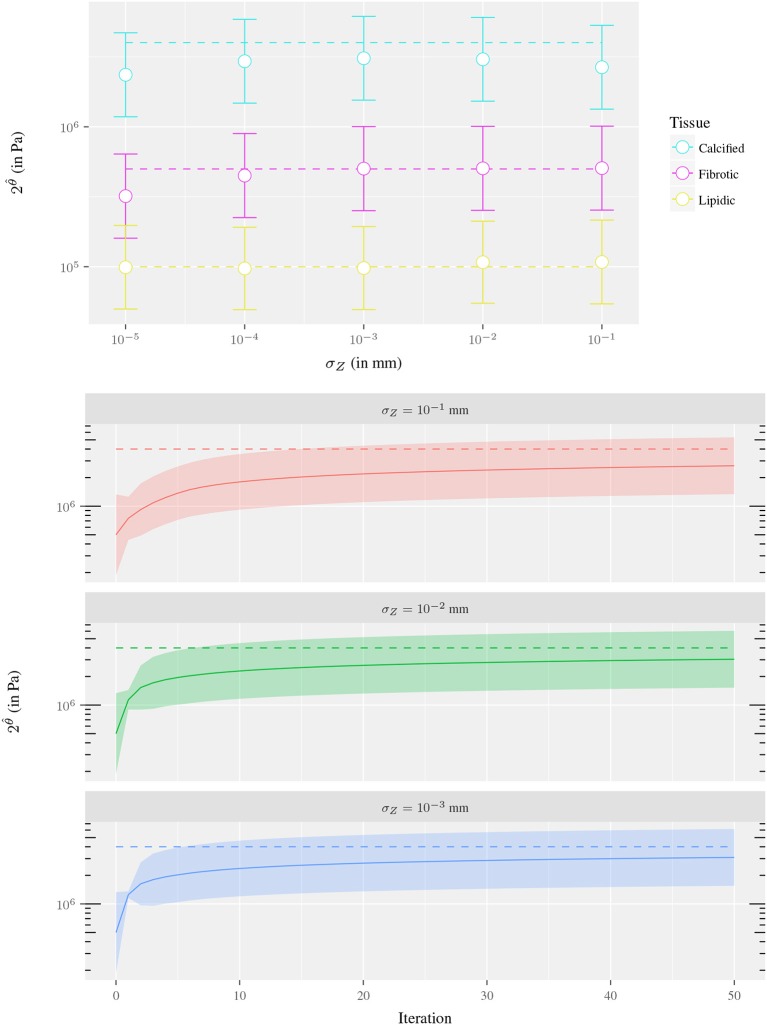
Sensitivity analysis of the parameter estimation with respect to σ_*Z*_ for the experiment of the 1-material ring fixing the parameter uncertainty σ_θ_ = 2: **(Top)** Estimate using observation uncertainties of $\sigma_{Z}=10^{i}$ mm, *i* = −5, …, −1 where each dot corresponds to a data assimilation process (after 200 iterations). The color indicates the material of the ring at each experiment, the dashed line is *c*^*t*^ value and the whiskers denote the parameter uncertainty interval. **(Bottom)** Convergence of the Kalman filter using observation uncertainties of $\sigma_{Z}=10^{i}$ mm, *i* = −3, −2, −1 for *c*^*t*^ = 4·10^−6^ Pa. The dashed line is *c*^*t*^ value, the solid line the Kalman filter estimate $2^{\hat{\theta }}$ and the colored ribbon denotes the parameter uncertainty interval. In both cases, the uncertainty interval is estimated as $\left[2^{\hat{\theta }-\sqrt{\text{diag}(U^{-1})}};2^{\hat{\theta }+\sqrt{\text{diag}(U^{-1})}}\right]$.

To analyze the sensitivity of $\hat{\theta }$ with respect to the observation uncertainty σ_*Z*_, the estimation of the parameter is performed assuming different values σ_*Z*_, ranging from 10^−1^ to 10^−5^ mm. Also, three different materials are used for the ring, mimicking: cellular fibrotic tissue (*c*^*t*^ = 5·10^5^Pa), lipidic tissue (*c*^*t*^ = 1·10^5^Pa) and calcified tissue (*c*^*t*^ = 4·10^6^Pa). The estimation of the Kalman filter for all the 15 cases is presented in Figure 5. The results showed that in all cases the parameter uncertainty interval $\left[2^{\hat{\theta }-\sqrt{\text{diag}\left(U^{-1}\right)}};2^{\hat{\theta }+\sqrt{\text{diag}\left(\left(U^{-1}\right)\right)}}\right]$ encloses the true parameter value *c*^*t*^. Even though, a closer estimate across the three materials is obtained for $\sigma_{Z}=10^{-3}$ mm which seems reasonable as it is the precision of the displacements delivered by the convergence process in solving the nonlinear operator *f*.

Regarding the filter convergence, it is observed that as the uncertainty in the observations decreases, the method converges faster. In Figure 5, it is shown that as the σ_*Z*_ increases its value, the convergence is slower. Note that the estimator gain matrix is computed as ${\text{K}}_{k}={\text{L}}_{k}^{\theta }{\text{U}}_{k}^{-1}{\left\{\text{H}\text{L}\right\}}_{k}^{T}{\text{R}}_{k}^{-1}$ and the only operator that varies in the first iteration of the presented cases is ${\text{R}}_{0}^{-1}$. As the spectral radius of ${\text{R}}_{0}^{-1}$ diminishes as σ_*Z*_ increases then **K**_0_ spectral radius diminishes as well, yielding a smaller correction of ${\hat{\theta }}_{k}^{+}$ as presented in the plot. At the same time, since **P**_*w*_ is constant, the update of ${\text{U}}_{k}={\text{P}}_{w}+{\left\{\text{H}\text{L}\right\}}_{k}^{T}{\text{R}}_{k}^{-1}{\left\{\text{H}\text{L}\right\}}_{k}$ is damped by **R**_*k*_. This damping effect is evidenced in the evolution of the parameter uncertainty intervals plotted in Figure 5. In statistical terms, the lack of confidence in the new observations leads to reducing its weight at the correction step.

**Figure 6:**
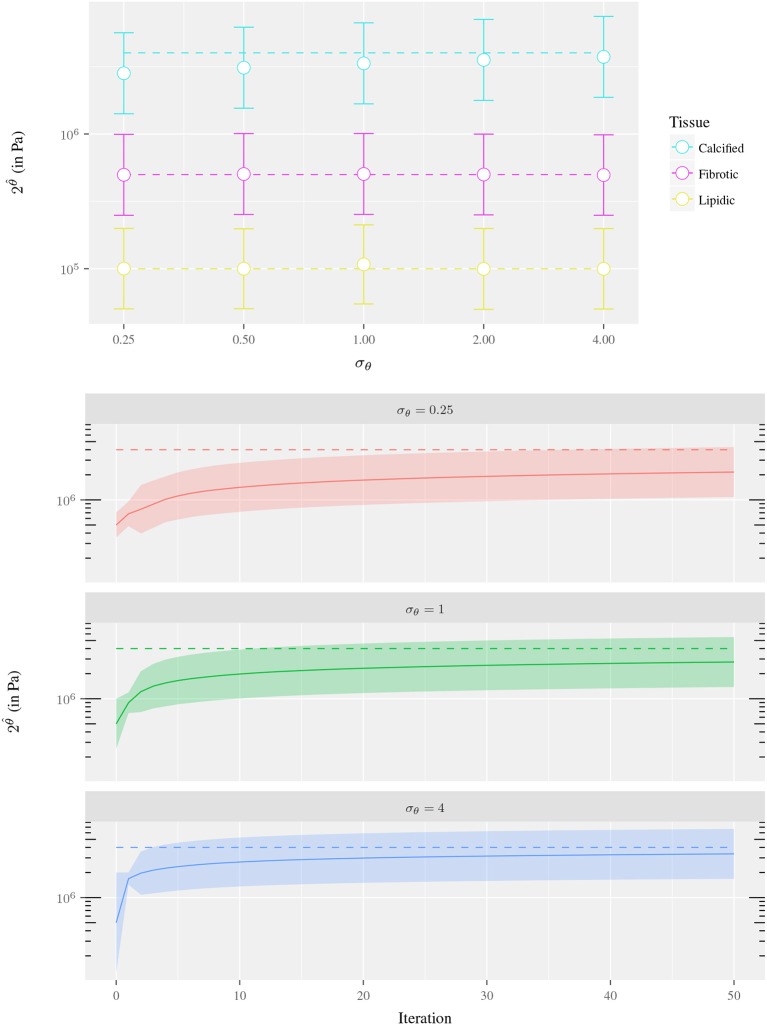
Sensitivity analysis of the parameter estimation with respect to σ_θ_ for the experiment of the 1-material ring fixing the parameter uncertainty $\sigma_{Z}=10^{-2}$: **(Top)** Estimate using parameter uncertainties of σ_θ_ = 0.25, 0.5, 1, 2, 4 where each dot corresponds to a data assimilation process (after 200 iterations). The color indicates the material of the ring at each experiment, the dashed line is *c*^*t*^ value and the whiskers denote the parameter uncertainty interval. **(Bottom)** Convergence of the Kalman filter using parameter uncertainties of σ_θ_ = 0.25, 1, 4 and *c*^*t*^ = 4·10^6^ Pa. The dashed line is *c*^*t*^ value, the solid line the Kalman filter estimate $2^{\hat{\theta }}$ and the colored ribbon denotes the parameter uncertainty interval. In both cases, the uncertainty interval is estimated as $\left[2^{\hat{\theta }-\sqrt{\text{diag}(U^{-1})}};2^{\hat{\theta }+\sqrt{\text{diag}(U^{-1})}}\right]$.

**Figure 7:**
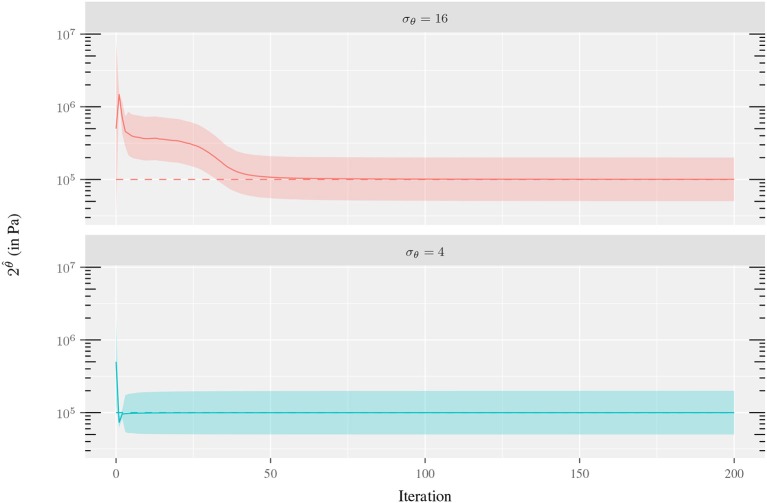
Convergence of the Kalman filter for the experiment of the 1-material ring using σ_θ_ = 4, 16, $\sigma_{Z}=10^{-2}$ mm and 2^θ^*t*^^ = 1·10^5^ Pa. The dashed line is 2^θ^*t*^^ value, the solid line the Kalman filter estimate $\hat{\theta }$ and the colored ribbon denotes the parameter uncertainty interval $\left[2^{\hat{\theta }-\sqrt{\text{diag}(U^{-1})}};2^{\hat{\theta }+\sqrt{\text{diag}(U^{-1})}}\right]$.

An analogous analysis was performed to study the sensitivity of $\hat{\theta }$ with respect to the parameter uncertainty σ_θ_. The uncertainty levels for σ_θ_ ranged from 0.25 to 4 and the experiment was repeated for the three different ring materials (fibrotic, lipidic and calcified tissues). The results showed that the bigger σ_θ_, the wider the search space for the parameter, and the faster the method converges when the initial value is far from the true parameter value (see Figure 6). On the other hand, high values of σ_θ_ may cause an overshooting in the estimation and a slower convergence. In this scenario, the reparametrization deteriorates the convergence even more. The reparametrization imposes an estimation bias to stiffer values due to the fact that displacements are less sensitive with respect to small variations in stiffer than softer materials. Then, the mean observation error (used as correction term in Equation 30) is biased to the sigma points associated with stiffer materials. This is clearly evidenced in Figure 7, where the initial overshooting delays the estimation of the parameter. Hence, note that the initial uncertainty interval does not necessarily has to contain *c*^*t*^ to estimate its correct value. In fact, the uncertainty parameter values are also iteratively updated and similar values are obtained for all three σ_θ_ illustrated in Figure 6. The role of the initial value of σ_θ_ is the dispersion of sigma points around the mean initial guess, and large values may accelerate convergence when the initial guess *c*_*i*_ is far from *c*^*t*^.

Overall, a good agreement is found in term of accuracy and convergence for parameters $\sigma_{Z}=10^{-3}$ mm and σ_θ_ = 4. These parameters identify clearly the three different kinds of tissues in this idealized problem. Also, the observations generated *in-silico* present an accuracy of similar order than the obtained (assuming no error carried by the optical flow) through the IVUS image processing. For this reason, $\sigma_{Z}=10^{-3}$ mm is used in cases analyzed in forthcoming sections. The value of σ_θ_ cannot be straightforwardly assigned because parameter overshooting using *in-vivo* complex geometries may lead to excessively soft materials which could cause contact at the inner surface in when solving the preload equilibrium, yielding non-free material configurations. Since stress-free configurations have been assumed, a more conservative value of σ_θ_ = 0.5 is used to avoid such problem.

### 3.2. Boundary conditions sensitivity

As described in section 2.2.1, the observational datum **u**^OF^ is considered as an additional information over ∂Ω^*E*^ through a penalization factor τ (i.e., a Robin boundary condition). This strategy is an attempt to incorporate the contribution of surrounding tissues through a surrogate surface model. Moreover, since **u**^OF^ can be exposed to errors caused by brightness variations, image artifacts or non-physical optical flow regularization issues, the use of a Robin boundary condition allows the model to naturally filter out the field **u**^OF^ similarly as a surface spring model. Then, a characterization of the surrounding tissues provided by τ in the parameter estimation is addressed in this section.

**Figure 8:**
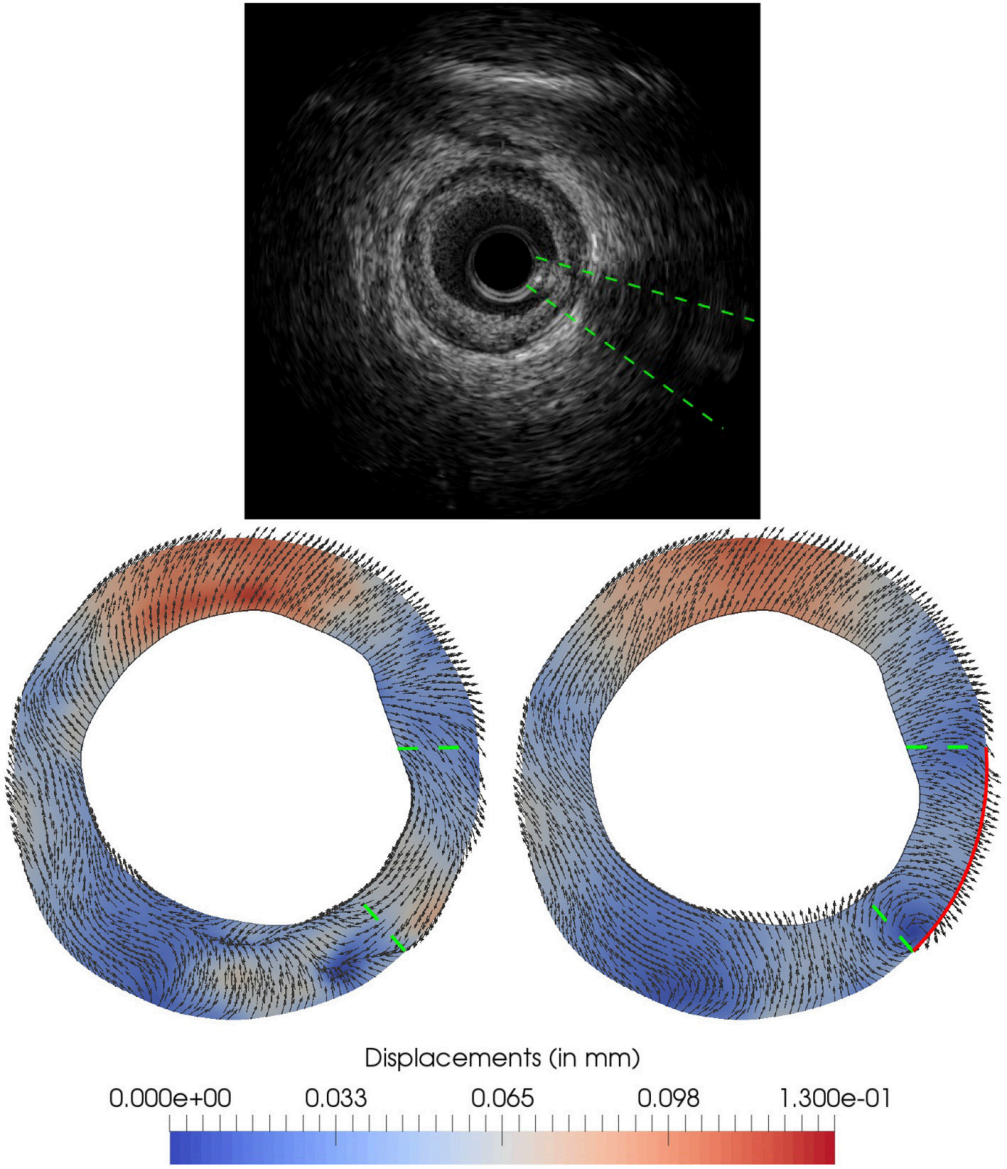
Optical flow and proposed ground truth for *in-silico* test: **(Top)** IVUS image at end-diastolic phase; **(Bottom-left)** optical flow **u**^OF^ used in the data assimilation process extracted from the *in-vivo* IVUS pair of images between end-diastole and systole phases; **(Bottom-right)** proposed ground truth ${\text{u}}_{2}^{\text{GT}}$ generated using homogeneous Neumann boundary condition over the solid red line. The green dashed line indicates the position of the guidewire artifact in the image, and therefore the area in which the optical flow displacement field can be largely affected.

The *in-silico* study case used for this sensitivity analysis was generated from the cross-section IVUS image depicted in Figure 8 by considering the configurations corresponding to two cardiac phases: end-diastole and systole. The geometrical model was constructed for the end-diastolic configuration following the pipeline described in sections 2.1 and 2.2.5. The configurations at each one of the cardiac phases are related to an end-diastolic load (i.e., the preload) of *t*^*W, n*^ = 80mmHg and to a systolic load *t*^*W, n*^ = 120mmHg, accordingly. The loads are applied over the inner surface of the vessel in the preload and forward problems, respectively. Finally, tethering tractions ${\text{t}}_{s}^{A,i}$ are considered such that an axial stretch of 10% is prescribed in the end-diastolic configuration. The remaining setup of boundary conditions is defined for each of the following analyses: (i) parameter estimation sensitivity as τ decreases from a large value (almost Dirichlet condition) to a small value (almost Neumann condition); (ii) parameter estimation robustness when observation **u**^OF^ features errors at the boundaries.

#### 3.2.1. Test 1: sensitivity of τ for error-free observations

For this analysis, the observations for the ROUKF were generated by solving the mechanical equilibrium with our model, avoiding observational and modeling errors. Thus, a Robin boundary condition was imposed at the outer surface in the forward problem with τ = 10^6^ (practically yielding a Dirichlet boundary condition). This setting rendered a ground truth displacement field ${\text{u}}_{1}^{\text{GT}}$ for this test. Using observations ${\text{u}}_{1}^{\text{GT}}$, the data assimlation algorithm was executed for τ∈{10^6^, 10^4^, 10^2^} (higher values of τ were not analyzed since τ = 10^6^ is already almost a Dirichlet boundary condition). The geometric model was partitioned in sextants with two concentric layer yielding 12 regions each with its own material parameter *c*_*i*_.

**Figure 9:**
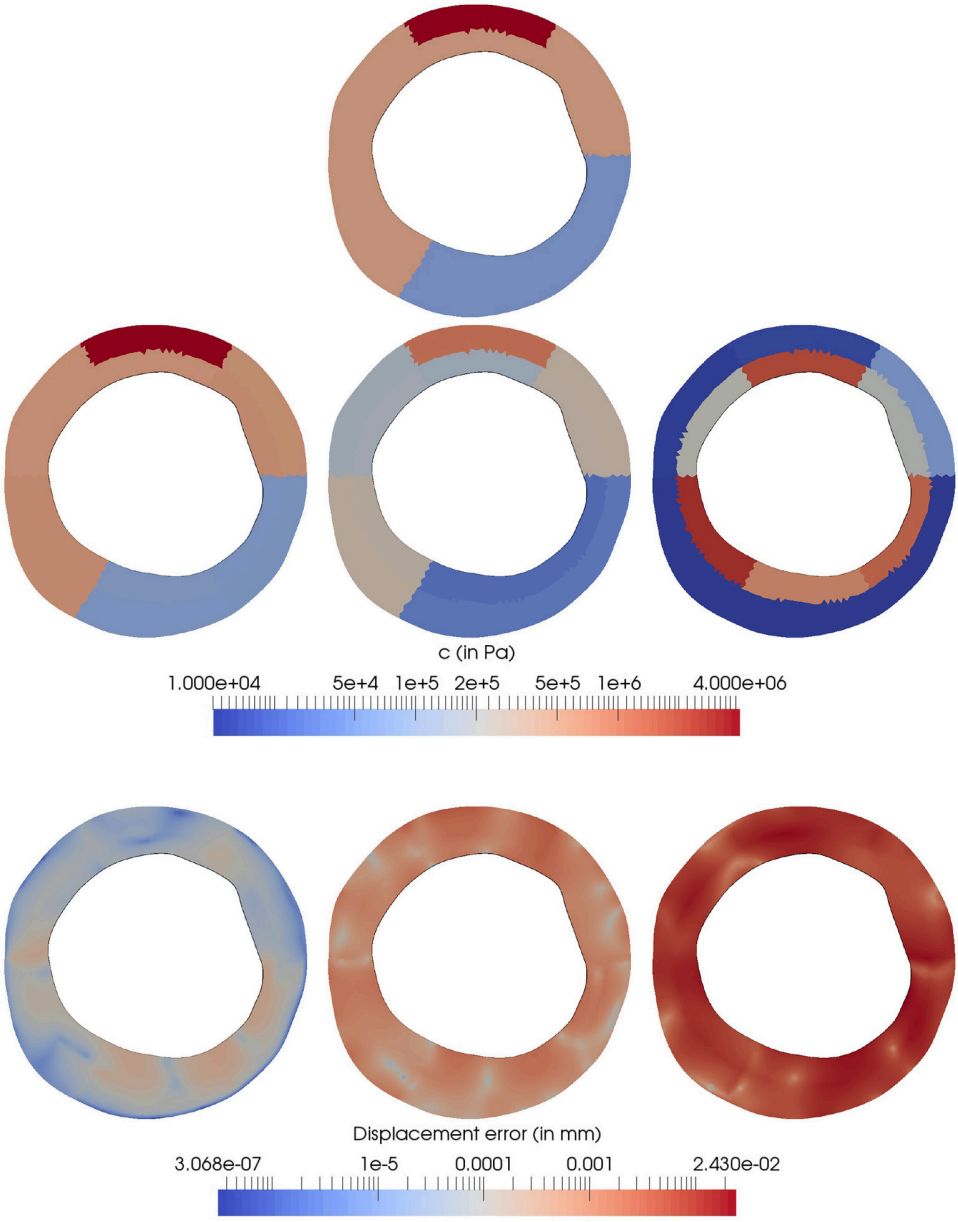
Sensitivity of the parameter estimation with respect to the penalization factor τ. **(Top)** *In-silico* model with the ground truth *c*^*t*^ parameters and boundary condition model with τ = 10^6^ for the generation of $Z=h\left(f\left(\left[0_{\text{u}},0_{\lambda },\theta^{t}\right]\right)\right)$. **(Middle)** Estimated parameters $\hat{\theta }$ from the observation *Z* by using τ = 10^6^, 10^4^, 10^2^ (from left to right) in the forward operator *f* during the data assimilation process. **(Bottom)** Displacement error (ε_*Z*_ = *Z*−Ẑ_*k*_ mm) for the parameter estimation process using τ = 10^6^, 10^4^, 10^2^ (from left to right) in the forward operator *f*.

The results are presented in Figure 9, depicting the parameter estimation and predicted observations variations as the Robin boundary condition moves toward a Neumann boundary condition. The decrease of forces at the boundaries caused by the decreasing value of τ is compensated by the estimation of softer materials (which experiment higher strains) to match the ${\text{u}}_{1}^{\text{GT}}$ observations. Particularly, the method recovers the correct material parameters when the penalization value is the true value used to generate the observations. i.e., τ = 10^6^. For the parameter estimation with τ = 10^4^, a qualitatively similar distribution of materials is observed with an uniform reduction in the magnitude of the material parameter. The lowest penalization value, τ = 10^2^, delivers a totally different arrangement of materials. This result emphasizes the important contribution of the surrounding tissues for a correct estimation of material parameters, which is clearly retrieved when sufficient large values of the penalization parameter τ are employed.

The observation error |ε_*Z*_|, which is defined as the Euclidean distance between the observations *Z* and mean filter observation Ẑ_*k*_ at the last iteration, increases as τ is decreased. Specifically, the mean values of ε_*Z*_ are 7.16·10^−5^, 1.03·10^−3^ and 9.79·10^−3^ for τ = 10^6^, 10^4^ and 10^2^, respectively. Clearly, should the observations ${\text{u}}_{1}^{\text{GT}}$ be error-free at the boundaries, a Dirichlet boundary condition (a higher value for τ) would be the correct choice. Notwithstanding this, the observations from *in-vivo* scenarios are degraded by diverse sources of errors and, as it will be shown next, an excessively stringent boundary condition (of Dirichlet type) may not be the best option.

#### 3.2.2. Test 2: sensitivity of τ for realistic observations

The current analysis aims at assessing the robustness of the parameter estimation process when the **u**^OF^ at the outer boundary differs from the real *in-vivo* displacements. For this purpose, an allegedly ground truth ${\text{u}}_{2}^{\text{GT}}$ is generated by altering the observation **u**^OF^ in a certain region (untrusted region) using the mechanical model with Neumann conditions. Finally, the assimilation process is performed with the observation **u**^OF^ and different values of τ, to assess if it is capable to approximate ${\text{u}}_{2}^{\text{GT}}$ despite the observation errors.

Thus, an IVUS sequence with a swinging artifact (induced by the guidewire) was chosen to perform our analysis. The IVUS cross-section depicted in Figure 8 presents an image artifact from the IVUS guidewire at the bottom-right quadrant of the frame. The guidewire projects a shadow that hides the arterial wall and, as consequence, the optical flow is polluted with a swinging movement not related with the true arterial-wall motion. Thus, a displacement field, denoted by ${\text{u}}_{2}^{\text{GT}}$, is generated from the *in-vivo* data removing the guidewire influence, with the purpose of comparing this ground truth against the Kalman predictions Ẑ_*k*_ when the polluted optical flow **u**^OF^ is used as observations. In that manner, the difference $\varepsilon^{\text{GT}}={Ẑ}_{k}-{\text{u}}_{2}^{\text{GT}}$ can be regarded as an estimate of the error in the Kalman prediction due to the artifact in the image processing data. At last, ε^GT^ is computed for different values of τ to assess the discrepancies in the predictions as the external Robin boundary condition is characterized differently.

The displacement ${\text{u}}_{2}^{\text{GT}}$ is generated by solving the equilibrium problems with a model constituted by a single material. To define a reasonable value for this constitutive property, a data assimilation process was performed using **u**^OF^ as observation and τ = 10^4^, yielding to *c* = 33.52 kPa. Note that the *c* is biased by the image artifact among other errors in the displacement field and it cannot be regarded as an estimate of the real material, thus, it is analyzed the ranges among which the estimated ĉ varies. At the boundary $\partial \Omega_{m}^{E}$, a Neumann homogeneous condition (τ = 0) was applied in the area affected by the guidewire (see red line in Figure 8) and a Robin boundary condition with τ = 10^4^ was applied to the remaining part of the boundary. The obtained displacement field ${\text{u}}_{2}^{\text{GT}}$ is displayed in Figure 8.

**Figure 10:**
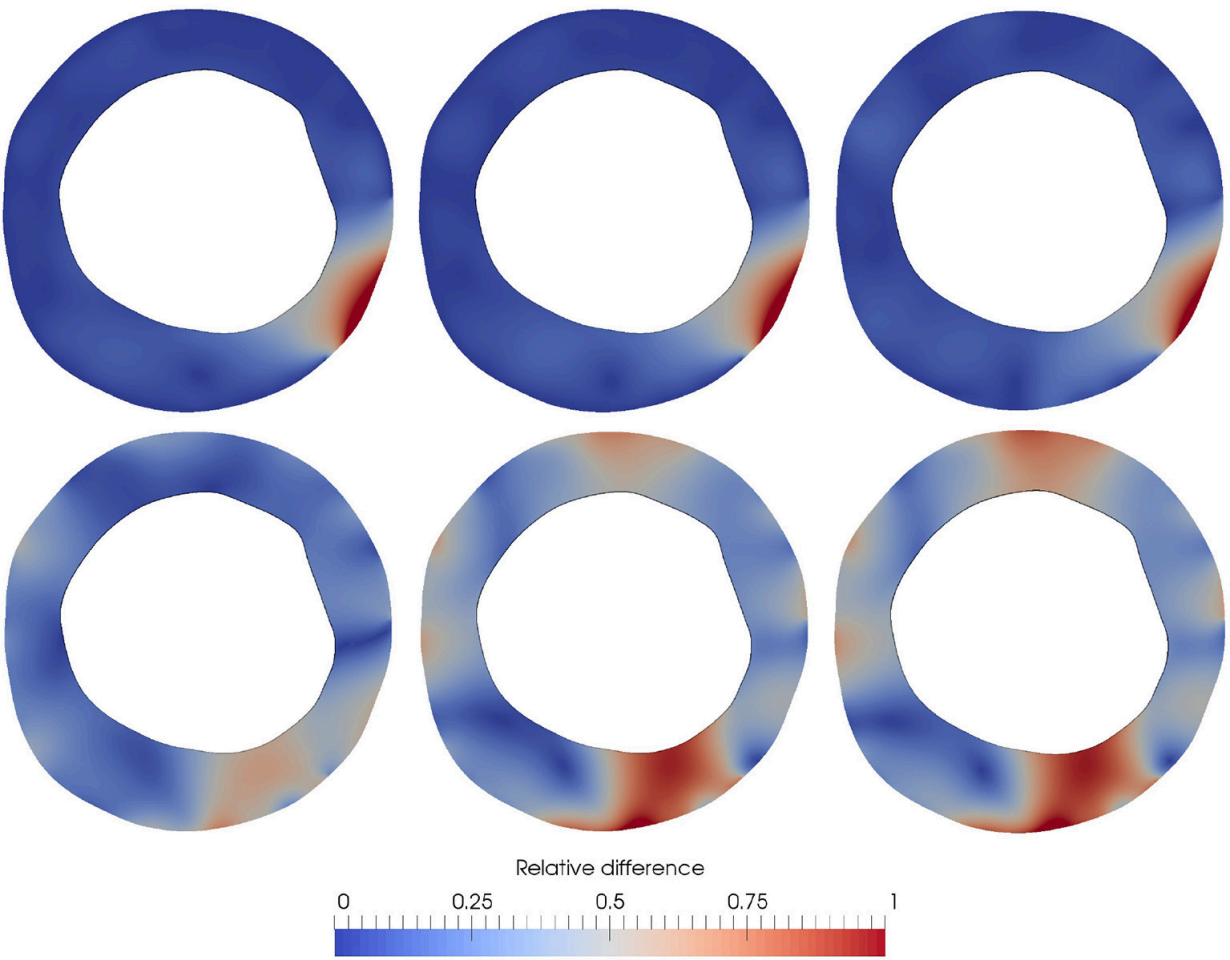
Relative difference $\varepsilon_{r}^{\text{GT}}=\frac{\varepsilon^{\text{GT}}}{\langle ∥{\text{u}}_{2}^{\text{GT}}∥\rangle }$ with respect to the boundary parameter τ. 〈·〉 Denotes the mean value in Ω_*s*_. From top-to-bottom and left-to-right, $\varepsilon_{r}^{\text{GT}}$ is presented for τ = 10^5^, 10^4^, …, 10^0^.The estimation of *c* was of 123.96, 33.52, 27.93, 68.51, 144.88 and 125.04 kPa for τ = 10^5^, 10^4^, 10^3^, 10^2^, 10^1^, and 10^0^ respectively, while the value of *c* in the generated ground truth is *c* = 33.52 kPa.

The sensitivity of ε^GT^ with respect to τ is then studied. For each value of τ ∈ {10^*i*^, *i* = 5, 4, …, 0}, the data assimilation process is executed using **u**^OF^ as observation. The relative difference between the generated ground truth ${\text{u}}_{2}^{\text{GT}}$ and the Kalman predicted observation *Z*_*k*_, for each τ, is reported in Figure 10. For τ greater than 10^3^, the Robin condition guarantees that the artifact-related displacements are preserved regardless the impact on the induced internal stresses. When τ varies from 10^3^ to 10^2^, the relative error difference significantly drops at the guidewire locus, from 1.33 to 0.58. As τ decreases even more, the resulting force induced by the Robin boundary condition diminishes its magnitude, yielding lower internal stresses, and spreading the error outside the region of the guidewire shadow. For values lower than 10^2^, the error in the displacement field is concentrated at the bottom area of the artery. Particularly, this concentration of the error is explained by the fact the continuum model is enforced to behave as incompressible, while the optical flow is not divergence-free. In terms of the parameter estimation, the value of *c* was of 123.96, 33.52, 27.93, 68.51, 144.88, and 125.04 kPa for τ = 10^5^, 10^4^, 10^3^, 10^2^, 10^1^, and 10^0^ respectively, presenting mean and standard deviation value of 87.31±50.70 kPa, all close to a cellular fibrotic tissue. Moreover, there is a large sensitivity in the estimated parameter with respect to the chosen value of τ. In comparison with the ground truth, the closest matching prediction in terms of the displacement field (i.e., the prediction for τ = 10^2^, see Figure 10) presents an estimation of *c* two times higher. This is a clear demonstration of the large sensitivity in the estimated parameter with respect to the setting of models for the external tissues. Even more, it indicates that the minimization of the displacement field is not directly related to the best parameter estimation.

### 3.3. Effects of preload and axial stretch

An appropriate baseline stress state of the vessel is key toward an accurate characterization of the stress state in arterial tissues. In fact, as reported in Ares (2016), a preloaded and axially stretched artery features notoriously different stress patterns compared to the case when such loads are neglected. Therefore, it is important to quantify the change in the parameter estimation when the initial stress state is either considered or not in the analysis. To quantify such disagreement, the parameters of an *in-vivo* study were estimated assuming three different conditions, namely: (i) the diastolic configuration is neither preloaded nor axially stretched; (ii) the diastolic configuration is preloaded but not axially stretched; (iii) the diastolic configuration is preloaded and 5% axially stretched; and (iv) the diastolic configuration is preloaded and 10% axially stretched. The choice for the last two cases is based on the experimental observations of Holzapfel et al. (2005) where it is reported a physiological range for axial stretch in coronary arteries ranging between 5 and 10%.

The geometrical model and the optical flow **u**^OF^ used for this study are the ones previously presented in Figure 8. The geometric model was partitioned in sextants with a unique concentric layer leading to the estimation of 6 material parameters (the same partition used in **Figure 12**. The remaining parameters for the mechanical problems and data assimilation process are described in Table 1 along with the estimated values *c*_*i*_. The results showed different trends for soft (*c* < 200 kPa, i.e., *c*_1_, *c*_2_, *c*_3_, and *c*_5_) and stiff materials (*c* ≥ 200 kPa, i.e., *c*_4_ and *c*_6_). The obtained parameter *c* increases in the soft tissues and decreases in stiff tissues as the baseline stress increases from a preload-free to a preloaded state. Further increments in the baseline stress due to the axial stretch result in material stiffening for these two categories of tissues. Interestingly, the increment of the parameter uncertainty σ_θ_ or the decrement of the observation uncertainty σ_*Z*_ in the stiff tissues increases the estimate of parameter *c* more in the preload-free state than in the preloaded cases. In fact, in the cases 2 and 3, the preloaded and 5% axially stretched model (cases 2.C and 3.C) featured lower *c* values in the stiff tissues than the preload-free model (cases 2.A and 3.A), contrarily to case 1. Some of these findings may appear counter-intuitive at first glance because as the baseline stress state increases it would be expected that all tissues soften to maintain the same deformation for the given load. Thus, the following paragraphs address the role of assimilation uncertainties, image artifacts, and the very mechanical model in the assimilation.

**Table 1:** Sensitivity of the parameter estimation with respect to the baseline stress conditions and uncertainty parameters.

**Case**	**σ_θ_**	**σ_*Z*_**	**Preloaded**	**Axial**	**Estimated parameters (in kPa)**						**ε_*Z*_**
		**(in mm)**		**stretch%**	***c*_1_**	***c*_2_**	***c*_3_**	***c*_4_**	***c*_5_**	***c*_6_**	
1.A	1	10^−2^	No	0	53	26	30	394	45	578	0.1562
1.B	1	10^−2^	Yes	0	59	29	37	384	51	582	0.1571
1.C	1	10^−2^	Yes	5	61	30	39	409	53	630	0.1572
1.D	1	10^−2^	Yes	10	65	32	41	413	59	634	0.1573
2.A	4	10^−2^	No	0	51	25	30	467	40	664	0.1562
2.B	4	10^−2^	Yes	0	57	28	37	442	48	620	0.1571
2.C	4	10^−2^	Yes	5	60	30	39	456	51	648	0.1572
2.D	4	10^−2^	Yes	10	63	32	41	476	55	676	0.1573
3.A	1	10^−3^	No	0	51	25	30	412	40	694	0.1562
3.B	1	10^−3^	Yes	0	58	28	37	394	48	669	0.1571
3.C	1	10^−3^	Yes	5	60	29	39	408	51	695	0.1572
3.D	1	10^−3^	Yes	10	63	31	41	424	55	721	0.1573

*In all cases, the boundary condition was fixed with τ = 10^2^ and the initial guess for the parameters was ${\hat{\theta }}_{0}^{+}=\left[c_{0},\ldots ,c_{\text{6}}\right]$ with c_i_ = 500 kPa ∀i. The estimated parameters are reported for each case, as well as the observation error |ε_Z_| = |Z−Ẑ_k_| after the data assimilation process*.

Firstly, as the baseline stress at the diastolic configuration rises, the parameter estimation is less sensitive with respect to variations between the predicted and the observed displacements i.e., *Z*−Ẑ_*k*_. For the different baseline stress states, it was assumed the same observation uncertainty which is analog to establish an uncertainty interval for the observed strains. As the Neo-Hookean model consists of a quadratic stress-strain relation, the increment of the baseline stress yields an increase in the uncertainty interval of the stresses. And because the stress is linear to the material parameter *c*, the estimated parameters undergo the same increase of their uncertainties diminishing the accuracy of their estimation. Moreover, the estimated value of *c* increased as the baseline state is subjected to a more significant preload condition, turning the data assimilation process even less sensitive. In short, this implies that dealing with the real problem –for which preload is definitely a condition of the vessels– is even more challenging than the case where initial stress conditions are neglected.

Secondly, the gap between the observations and the predicted displacements, hereafter simply *discrepancy*, in the data assimilation process is in part given by some observed displacement components generated by errors in the image processing stage and by physical phenomena which is not recoverable by the proposed mechanical and material models (e.g., external tissues, off-plane displacements, compressible materials or, even, misrepresentation of the constitutive law). These discrepancies could be referred to as out-of-model components, introducing a bias in the predicted displacement field and in the parameter estimation as well. Comparing the estimations with different baseline assumptions, it is observed that the discrepancies of the identified parameter value remain below 37% and 10% for soft and stiff tissues, respectively. Particularly, we choose to use the more complex model (preloaded and axially stretched) in the following *in-vivo* studies because it endows the mechanical setting with more relevant physical features when compared to the other models.

### 3.4. *In-vivo* cases

The proposed methodology is now applied to 4 *in-vivo* cases featuring atherosclerotic lesions to derive their specific mechanical models. The goal is to analyze the accuracy of the mechanical models to predict the optical flow observations, as well as, to assess the usage of multiple (more than two) cardiac phases (and then more than one optical flow displacement field as observational data) in the parameter estimation. For each lesion, the IVUS frames that are involved in the data assimilation correspond to end-diastole, 50%-systole and full-systole, as dictated by the ECG signal of the IVUS study. Optical flow was estimated between end-diastole and 50%-systole frames and end-diastole and full-systole frames, denoted by ${\text{u}}_{1}^{\text{OF}}$ and ${\text{u}}_{2}^{\text{OF}}$ respectively (see Figure 12). Then, we compare the resulting estimated parameter for two cases: when the assimilation is performed using a single optical flow displacement field as observation ($Z={\left[{\text{u}}_{2}^{\text{OF}}\right]}^{T}$); and when two optical flow displacement fields are utilized as observations ($Z={\left[{\text{u}}_{1}^{\text{OF}},{\text{u}}_{2}^{\text{OF}}\right]}^{T}$). Note that the observed displacement field for maximum load, i.e., ${\text{u}}_{2}^{\text{OF}}$, is employed in both cases because the displacement between end-diastole and systole is expected to yield higher strains.

**Figure 11:**
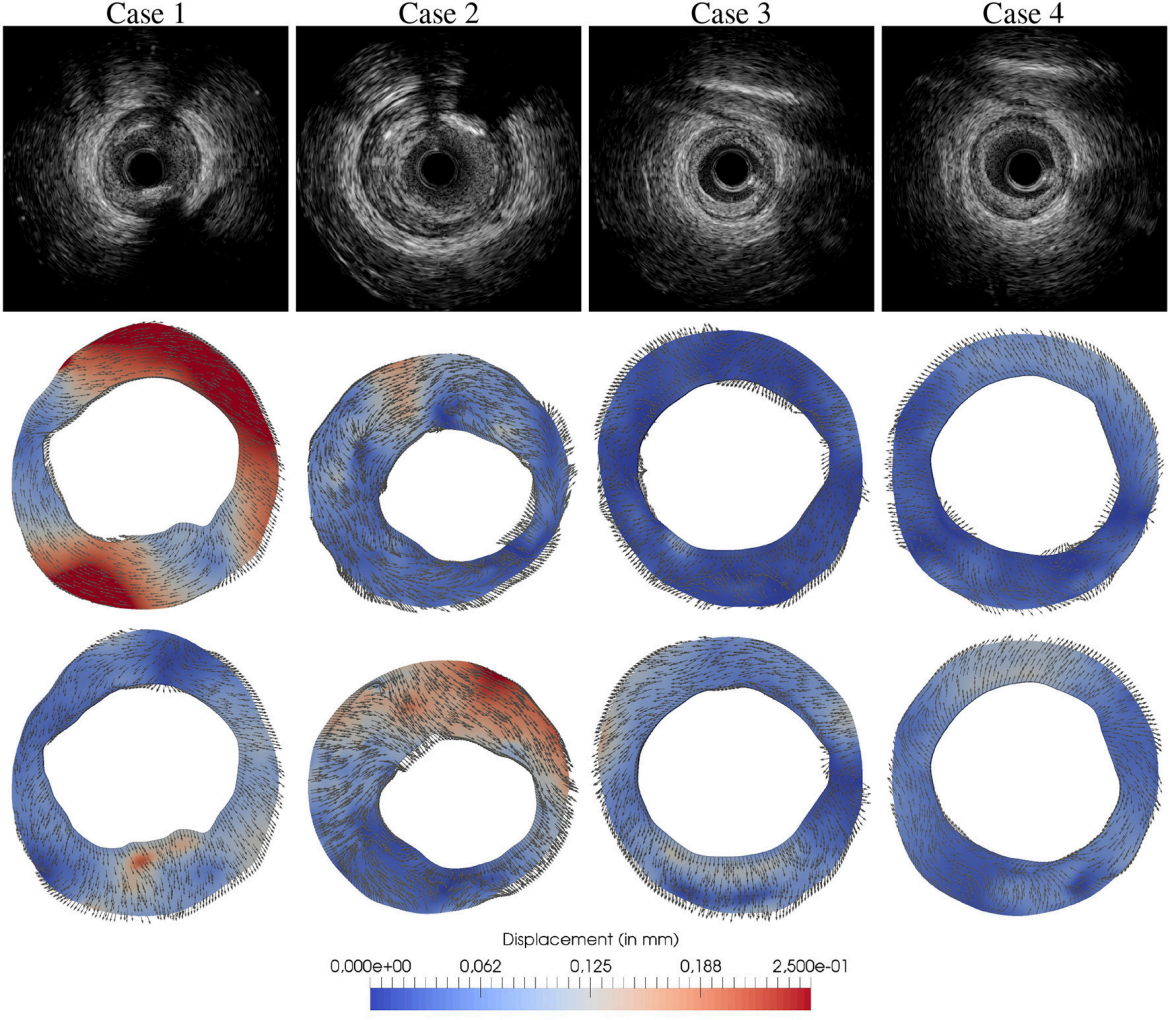
Optical flow estimated between end-diastole and 50%-systole frames, ${\text{u}}_{1}^{\text{OF}}$, and end-diastole and full-systole frames, ${\text{u}}_{2}^{\text{OF}}$ for 4 *in-vivo* atherosclerotic lesions: (row 1) IVUS end-diastolic frame depicting the atherosclerotic lesions; (row 2) displacement field ${\text{u}}_{1}^{\text{OF}}$; (row 3) displacement field ${\text{u}}_{2}^{\text{OF}}$.

The geometric model was partitioned in sextants with a unique concentric layer (see Figure 11). Each partition contains only a single type of material leading to a data assimilation process with 6 material parameters. The diastolic configuration is preloaded and 10% axially stretched for all cases and the blood pressure at each phase was assumed to be 80, 100, and 120 mmHg for the end-diastole, 50%-systole and full-systole, respectively. The parameter τ was set to 100 for lesions 1, 3, and 4 and 50 in case 2, the latter avoided contact at the luminar surfaces during in the preload problem. The ROUKF uncertainties were fixed to σ_θ_ = 1 and $\sigma_{Z}=10^{-2}$ mm.

**Figure 12:**
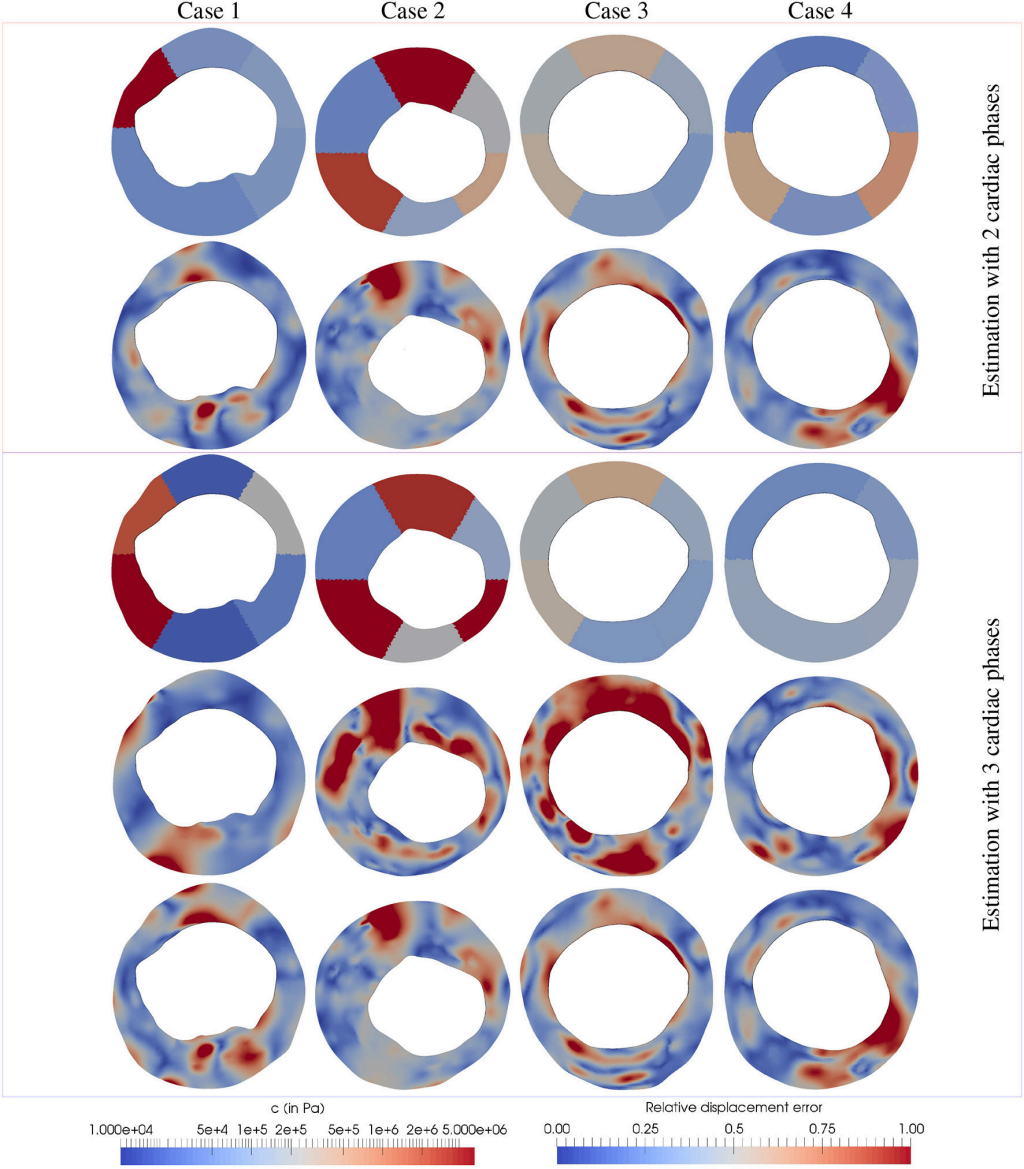
Parameter estimation and discrepancies between the model prediction and observations for 4 *in-vivo* IVUS frames featuring atherosclerotic plaques (one per column). (Row 1) Estimation of parameters *c*_*i*_ using $Z={\left[{\text{u}}_{2}^{\text{OF}}\right]}^{T}$; (row 2) ε_*r*_ for the observations related to ${\text{u}}_{2}^{\text{OF}}$; (row 3) Estimation of parameters *c*_*i*_ using $Z={\left[{\text{u}}_{1}^{\text{OF}},{\text{u}}_{2}^{\text{OF}}\right]}^{T}$; (row 4) ε_*r*_ for the observations related to ${\text{u}}_{1}^{\text{OF}}$; (row 5) ε_*r*_ for the observations related to ${\text{u}}_{2}^{\text{OF}}$. The relative displacement discrepancy between the model predictions and the observations was defined as ε_*r*_ = ∥Ẑ_*k*_−*Z*∥/〈∥*Z*∥〉 where Ẑ_*k*_ are the model predictions at the last Kalman iteration, *Z* are the optical flow observations and 〈·〉 denotes the mean value in Ω_*s*_.

The proposed data assimilation process rendered the results depicted in Figure 12. The material parameters estimated in all cases remained within the physiological range (between 1 kPa to 10 MPa, see Walsh et al., 2014). Also, the addition of an extra displacement field as observation showed no considerable effect for cases 2 and 3. The reliability of the results can be assessed in terms of the model prediction error presented in Table 2. Due to intrinsic sources of errors in the observations (motion artifacts, spatial incoherence between cross-sections in the cardiac cycle and optical flow model artifacts), it is expected an observation error of few pixel spacing units (recall that the image discretization spacing is 16μm). Thus, model prediction errors for cases 3 and 4, and even case 1 for a single optical flow field per cardiac phase, seems highly reliable in terms of our observation precision since the error results 26±14μm (1.625±0.875 pixel spacing units), while case 2 seems to be the less reliable estimation with an average error of 43±24μm. Overall, the average model prediction error was below 43μm and 61μm for the observation with one or two observational data, respectively.

**Table 2:** Model prediction error after data assimilation process for the 4 *in-vivo* cases using 1 or 2 loading conditions.

**Case**	**Amount of different loads (S)**	**Observation component $\left(Z_{k}^{j}\right)$**	**Model prediction error $Z^{j}-{Ẑ}_{k}^{j}$ (in mm)**			**Execution time (in hours)**
			**Mean**	**SD**	**Max**	
1	1	$Z_{k}^{1}$	0.023	0.014	0.122	18.32
2	1	$Z_{k}^{1}$	0.043	0.024	0.155	35.78
3	1	$Z_{k}^{1}$	0.026	0.014	0.094	12.20
4	1	$Z_{k}^{1}$	0.021	0.014	0.069	9.17
1	2	$Z_{k}^{1}$	0.061	0.016	0.271	33.37
		$Z_{k}^{2}$	0.029	0.041	0.113	
2	2	$Z_{k}^{1}$	0.032	0.022	0.111	77.37
		$Z_{k}^{2}$	0.043	0.024	0.155	
3	2	$Z_{k}^{1}$	0.012	0.008	0.071	41.07
		$Z_{k}^{2}$	0.026	0.014	0.094	
4	2	$Z_{k}^{1}$	0.015	0.010	0.064	16.39
		$Z_{k}^{2}$	0.021	0.015	0.074	

*The reported errors corresponds to the disagreement between mechanical model displacements (after estimate their material parameters with the proposed method) and the optical flow observations. The execution time corresponds to the wall clock time elapsed for each case using only mesh parallelism (see Figure 4) with 24 CPUs*.

In case 1, it is observed that the material parameters estimated with 1 and 2 optical flow displacement data are significantly different. The flow ${\text{u}}_{1}^{\text{OF}}$ presents larger displacements than ${\text{u}}_{2}^{\text{OF}}$, which seems counter-intuitive since the blood pressure variation is smaller for the former condition. However, the motion exerted by the cardiac contraction is higher, in fact the larger component of displacement is rigid (a rotation of the structures). Thus, as ${\text{u}}_{1}^{\text{OF}}$ presents the observation components with higher norm, it features a larger contribution than ${\text{u}}_{2}^{\text{OF}}$ during the data assimilation process (see Equation 30). In that manner, the parameters estimated with 1 flow datum minimize discrepancies against ${\text{u}}_{2}^{\text{OF}}$ while the ones estimated with 2 flow data minimize mainly discrepancies against ${\text{u}}_{1}^{\text{OF}}$.

Conversely in cases 2 and 3, the observation ${\text{u}}_{1}^{\text{OF}}$ is the one with smaller displacements (≈4 and 2 times smaller for cases 2 and 3, respectively), yielding a small contribution to the data assimilation. This implies that the minimization of the discrepancies between the model predictions and the observations (i.e., Ẑ_*k*_−*Z*) related to ${\text{u}}_{2}^{\text{OF}}$ dominates over the discrepancies associated to ${\text{u}}_{1}^{\text{OF}}$. In fact, Figure 12 shows that the discrepancies represented by ε_*r*_ for ${\text{u}}_{2}^{\text{OF}}$ remained almost invariant using 1 or 2 flows in the observation.

In case 4, the discrepancies between the model predictions and the observations related ${\text{u}}_{2}^{\text{OF}}$ also remained invariant using 1 and 2 flows data in the observation, although the parameters estimated in the lower part of the geometry varied significantly (see Figure 12). As previously studied in section 3.2.2, the guidewire artifact in the lower part of these images features a swinging movement not related with the arterial-wall motion. To approximate the artifact's rigid motion, the local tissue is stiffened during the assimilation process when 1 single flow was employed as observation. Conversely when ${\text{u}}_{1}^{\text{OF}}$ is added to the observations, the spurious motion of the guidewire is negligible, and the data assimilation is not affected by this artifact.

To determine the applicability of the current approach in clinical practice, we execute the *in-vivo* cases in a single server with 2 Intel Xeon CPU E5-2620 at 2.00 GHz processor (each with 12 threads) and Kingston 99U5471-031.A00LF at 1333 MHz (latency of 27 ns) RAM memory. For data assimilation of these *in-vivo* cases, mesh (3 threads per mechanical problem) and sigma parallelism (1 thread per sigma point) were applied because it delivered the best speed up for our 24 threads (actually only 21 were employed). The wall clock time reported in Table 2 for each execution showed that the current methodology is appropriate for offline medical applications because the processing times elapsed from 0.5 to 3 days. The use of clusters would allow further processing speed up exploiting the load parallelism as well as a more massive parallelization at the mesh level.

## 4. Discussions

The presented methodology offers a workflow to estimate material parameters for mechanical model of coronary arteries. The strategy is composed by three key components: the image processing, the mechanical model and the data assimilation algorithm. The most appealing aspect of this proposal is that the three components are loosely coupled as black boxes which allowed us to modify, as required, each component without the need for altering the remaining ones. In fact, the image processing renders observations for the data assimilation, regardless the imaging technique employed and the nature of the displacement field. In turn, the mechanical model can also be modified without influencing in the other components, it simply must receive a set of parameters and return back the internal state variables to the data assimilation strategy. Due to this architectural design, this initial biomechanical characterization approach can be further refined by improving aspects of these individual components. Some identified hot-spots for improvement are discussed in what follows.

The data assimilation showed high sensitivity with respect to variations in the model boundary conditions which aimed at mimicking the external tissues. As the displacement over the boundary was increasingly constrained (large τ) the model was less sensitive to variations in the material parameters, hindering the parameter estimation and, even, causing divergence of the Kalman iterative process in some situations. Also, the disagreement in the spatial arrangement of model forces and the *in-vivo* (unknown) forces at the boundary notoriously affects the outcome of the estimation. This was exposed in section 3.2.2 when an image artifact (the IVUS guidewire) induced a spurious tangential displacement in the observation and the boundary condition. It was also showed that if a homogeneous Neumann condition is assumed at the site of such artifact, the parameter estimation varies significantly (from 4 to 15 fold reduction of parameter *c*). Improving the capabilities of the model in this sense requires to incorporate the estimation of these forces exerted by external tissues in the data assimilation process. In short, parameter τ could be a further variable to be estimated.

It is also important to highlight that this approach can be directly extended to account for more geometrically and physically complex models. The set of here reported results constitute a solid proof of concept toward the extension of this methodology. Here, we derived a patient-specific mechanical model for an orthogonal slice of the vessel assuming plane strain state with an homogeneous axial traction force. However, there are some assumptions that imply neglecting certain physical components that may be necessary to increase the accuracy of the estimated stress/strain state of the vessel. To list some of them: (i) shear forces exerted by the blood flow which are expected to be key in the study of plaque development (Stone et al., 2003; Chatzizisis et al., 2008); (ii) out-of-plane forces produced by the blood pressure due to the heterogeneous constitution of the vessel wall and the tilting of the transducer tip with respect to the cross-section; and (iii) variable axial tractions along the cross-section due to the heterogeneous composition of the vessel wall. These issues can be tackled at once by making use of 3D models. In fact, the image processing strategy allows the gating and registration of the whole arterial 3D volume of the study. Also, the extension of the optical flow techniques to 3D domains is straightforward by a proper adaptation of the differential operators and Gaussian kernel within the formulation. A further issue to address is the spatial reconstruction to obtain the proper 3D geometrical description of the vessel instead of its rectified representation in intrinsic coordinates delivered by the IVUS study. The integration of IVUS with angiographic images enabled us to perform such 3D reconstruction, as reported in Maso Talou (2013). These extensions imply in heavier computational cost and complementary implementation aspects, yet, they present no further conceptual differences regarding the methodology presented in this work.

Extension to 3D problems discussed above, as said, becomes computationally more demanding. Associated to the image processing, the cost scales with the number of cross-sections extracted from the IVUS dataset. However, the registration stage, which is the most computationally intensive task, is fully parallel (see Maso Talou et al., 2017) and gating cost is negligible. Thus, the performance of the optical flow and the spatial reconstruction process through the integration with angiographic images, turn out to be key for the efficiency of the methodology in 3D cases. Regarding the data assimilation procedure, the computational cost continues to be the approximate solution of the mechanical problem. As a significant increase in the number of degrees of freedom is expected, the computational cost would raise as well.

A first limitation in the present scheme is that the displacement field retrieved from medical images is naively used as observation from our model without further processing. This implies that the performance of the method can be improved by extracting the observation components that are spurious (such as artifacts or unreliable regions of the optical flow displacement field) or even incompatible with our model (e.g., use only the divergence-free component of the field because the mechanical model is incompressible).

Regarding the baseline stress state in our model, the residual stresses produced during the arterial tissue genesis and growth have clearly been neglected. In Wang et al. (2017), an experimental test showed that the omission of these residual stresses may produce a significant overestimation of internal stresses (from 2- to 4-fold the actual stress). Furthermore, it has been observed (Guo et al., 2017) that accounting for residual stresses is also relevant for the proper material parameter estimation. This seems to be natural, as residual stresses can be considered as a subproduct of existing residual deformations (that, in general, may not be kinematically compatible, i.e., they cannot be derived from continuous displacement fields) of the elastin matrix. Consequently, not only the stresses are not properly assessed, but the actual deformations observed at the equilibrium states are misguided. These facts highlight the need for further research to tackle simultaneously the estimation of both material parameters and residual deformations in arterial walls. In recent works (Ares, 2016; Ares et al., 2017), models and methods for the estimation of such residual stresses were proposed, with a similar spirit to the one developed in this work.

At last, it is worthwhile to remark that no validation techniques are currently available for the assessment of stress-strain state in *in-vivo* conditions. Even though, approaches for an indirect *in-vivo* or *ex-vivo* validation can be discussed. Techniques such as elastography and palpography (Ophir et al., 1991; Shapo et al., 1996; Céspedes et al., 1997; de Korte et al., 1998; Céspedes et al., 2000) deliver with some degree of reliability the stresses in the innermost part of the vessel. In these cases a Bland-Altman analysis can be applied to assess the similarity between the prediction of our approach and elastographic solutions. A more controlled experimental setup can be planned for *ex-vivo* condition using coronary specimens. For each specimens, an IVUS study can be acquired and a specimen-specific model can be constructed employing the proposed methodology. Finally, several mechanical tests can be carried out with the specimens comparing their mechanical response with predictions given by our specimen-specific models. Another *in-vivo* alternative is to associate ranges of the estimated material parameters to the underlying tissue composition, delivering a histological description of the vessel (usually referred to as virtual histology). As there are already methods that estimate the vessel histology from IVUS images (e.g., Kawasaki et al., 2002; Nair et al., 2002; Sathyanarayana et al., 2009), a comparative analysis can be performed to evaluate the degree of agreement between the proposed method and these virtual histologies. An appealing aspect of this last validation is that the techniques presented in those works are already validated with cadaveric specimens of coronary arteries. The experimental settings suggested above should serve to bridge the world of computational models and methods with the experimental realm, toward gaining insight into the complex mechanisms underlying the development of cardiovascular diseases.

## 5. Final remarks

A data assimilation environment for analysis of arterial models and material characterization was described. The proposed methodology delivers the necessary tools to construct patient-specific mechanical models of an arterial site using data from standard IVUS studies. A complete sensitivity analysis of the biomechanical characterization with respect to numerical and physical parameters was reported to aid the methodology setup, as well as the interpretation of data assimilation outcomes. Validation in controlled scenarios was provided to demonstrate the capabilities of the present approach.

The potential and limitations of this approach were exposed and discussed in the previous section, delineating future research to enhance the image processing stage and the mechanical model of the arterial wall for this problem.

The applicability of this methodology on *in-vivo* scenarios was proven in the characterization of the arterial tissue for 4 *in-vivo* atherosclerotic lesions. After data assimilation, the obtained mechanical models predicted the displacement field between diastole and systole with errors below 43μm using frames of only two cardiac phases. Although no validation was performed with the *in-vivo* cases, the estimated material parameters remained within the expected range for this kind of tissue.

The development of this tool for the biomechanical analysis allows the indirect estimation of the internal stress state of the arterial wall. Such information combined with the vessel histology (that can be inferred from the material parameters here estimated) enables the assessment of the structural integrity of the atherosclerotic plaque to aid medical decisions and research. In summary the proposed strategy provides an imaging-assimilation-mechanics integrated environment to characterize, within a truly *in-vivo* and patient-specific setting, the behavior of the materials that compose the arterial vessels, specifically coronary vessels, which is of the utmost importance in assessing risk of plaque progress and rupture.

## Author contributions

GM, GA, PB, and RF designed the model and the computational framework. GM and GA carried out the computational implementation. GM, PB, and RF planned the experiments. GM and PB performed the calculations and wrote the manuscript with input from all authors. CG and PL performed measurements and contributed to sample preparation. All authors contributed to the discussion and interpretation of the results, and helped shape the final version of the manuscript.

### Conflict of interest statement

The authors declare that the research was conducted in the absence of any commercial or financial relationships that could be construed as a potential conflict of interest.

## Footnotes

^1^The reparametrization $2^{\hat{\theta }}$ modifies the assumed distribution of the parameter from normal distribution to log-normal distribution. Mean and covariance of θ are propagated in a different fashion than mean and covariance of *c*. Nevertheless, there is a similar statistical interpretation for *c* using these descriptors in an exponential space of coordinates. For example, a covariance of σ = 1 must be understood as giving the same probability of *c* being half or twice its initial value.
